# CSCN: inference of cell-specific causal networks using single-cell RNA-seq data

**DOI:** 10.1093/bioinformatics/btag480

**Published:** 2026-06-30

**Authors:** Menghan Wang, Junya Yang, Luyao Lyu, Jiaxing Chen

**Affiliations:** Faculty of Science and Technology, Beijing Normal-Hong Kong Baptist University, Zhuhai 519087, China; Faculty of Science and Technology, Beijing Normal-Hong Kong Baptist University, Zhuhai 519087, China; Faculty of Science and Technology, Beijing Normal-Hong Kong Baptist University, Zhuhai 519087, China; Guangdong Provincial/Zhuhai Key Laboratory IRADS, Beijing Normal-Hong Kong Baptist University, Zhuhai 519087, China

## Abstract

**Motivation:**

Understanding gene regulation is fundamental to deciphering the coordinated activity of genes within cells. Although single-cell RNA sequencing (scRNA-seq) enables gene expression profiling at cellular resolution, most gene network inference methods operate at the tissue or population level, thereby overlooking regulatory heterogeneity across individual cells. Recent approaches, such as Cell-Specific Network (CSN) and its extension c-CSN, attempt to construct gene networks at single-cell resolution, providing a more detailed view of the regulatory logic underlying individual cellular states. However, these methods remain limited by high false positive rates due to indirect associations and lack of directionality or causal interpretability.

**Results:**

To address these issues, we propose the Cell-Specific Causal Network (CSCN) framework, which infers directed, cell-specific gene regulatory relationships by explicitly modeling causality. CSCN combines causal discovery techniques with efficient computation using kd-trees and bitmap indexing to perform conditional independence testing, yielding sparse and interpretable causal graphs for each cell that effectively suppress indirect and spurious associations. Across nine scRNA-seq datasets, the Causal Katz Matrix (CKM) derived from CSCN provided more accurate and stable cell-state discrimination than expression-based and network-based baselines. CSCN-derived representations also preserved developmental structure, achieving the best trajectory performance in simulations and the strongest agreement with human embryo progression. Beyond RNA-only analysis, CSCN further generalized to paired PBMC multiome, CITE-seq, and spatial transcriptomic settings. Also, in controlled confounding simulations, CSCN consistently achieved the lowest false-positive rates relative to CSN and c-CSN.

**Availability and Implementation:**

The code is available at https://github.com/open17/CSCN.

## 1 Introduction

Understanding how genes interact to regulate cellular behavior is a fundamental goal in molecular biology. Gene-gene networks, such as gene regulatory networks (GRNs), provide a powerful framework to model the coordinated activities among genes. However, most existing methods construct these networks at the tissue or population level, implicitly assuming regulatory homogeneity across cells. Increasing evidence suggests that gene interaction patterns can vary significantly from cell to cell, influenced by intrinsic heterogeneity, developmental dynamics, and microenvironmental factors.

This realization has driven growing interest in constructing gene networks at single-cell resolution, aiming to capture the unique regulatory and functional landscape of each individual cell. Advances in single-cell RNA sequencing (scRNA-seq) (Bageritz and Raddi 2019, Juzenas *et al*. 2024) now enable gene expression profiling at the single-cell level, facilitating computational reconstruction of gene-gene networks on a per-cell basis. Gene networks at single-cell resolution offer a more detailed view of gene regulation by capturing the underlying control logic that shapes cellular functions. They provide critical insights into how individual cells interpret environmental signals, maintain distinct identities, and undergo state transitions. Compared to raw expression profiles, such network-based representations tend to be more stable and biologically informative, making them especially valuable for downstream single-cell analyses and for achieving a deeper mechanistic understanding of cellular heterogeneity.

Building on this foundation, the Cell-Specific Network (CSN) framework (Dai *et al.* 2019) was proposed to infer gene–gene association networks at single-cell resolution by capturing nonlinear dependencies and constructing the Network Degree Matrix (NDM) to replace raw expression data, thereby enhancing downstream analyses. However, since CSN relies solely on marginal independence tests, it cannot distinguish direct from indirect dependencies, often leading to false positives. For instance, two genes independently regulated by a shared transcription factor may appear connected despite lacking a direct interaction.

To distinguish direct and indirect dependencies, the conditional Cell-Specific Network (c-CSN) framework (Li *et al*. 2021) extends CSN by incorporating conditional independence tests thus reducing false positives in per-cell networks. While c-CSN improves specificity, it remains limited in two factors. First, its single-gene conditioning strategy fails to resolve indirect links when multiple regulators jointly control gene pairs—for example, genes A and B co-regulated by factors *X* and *Y* may still appear spuriously associated. Second, c-CSN produces undirected networks, lacking both edge directionality and causal interpretability. For example, it cannot differentiate whether gene A regulates gene B, vice versa, or if their relationship is reciprocal; all scenarios are treated as equivalent, potentially obscuring true regulatory direction and reducing the biological validity of downstream analyses.

To address these limitations, we propose the Cell-Specific Causal Network (CSCN) framework, a novel approach that, for the first time, infers causal gene regulatory networks at single-cell resolution. CSCN constructs directed graphs to represent gene regulatory interactions by explicitly modeling causality rather than relying on mere associations. This allows CSCN to eliminate indirect and spurious links, producing sparse, interpretable, and biologically meaningful networks in single-cell resolution. The resulting causal perspective offers a more accurate and robust representation of gene regulatory dynamics, enhancing downstream interpretability. CSCN adapts the causal inference algorithm Peter Clark (PC) (Pearl 2009) with gene-level conditional independence testing. To deal with the high computational demands of causal discovery, we incorporated kd-tree data structures and bitmap indexing to markedly accelerate the conditional independence testing for large-scale gene and cell data.

To evaluate the quality of the inferred causal networks, we perform clustering based on the Causal Katz Matrix (CKM), a representation derived from each cell-specific causal graph. CKM quantifies gene regulatory importance using Katz centrality, capturing both direct and multi-step causal influences while preserving the original expression matrix’s dimensionality for compatibility with standard downstream analyses. Across nine publicly available scRNA-seq datasets, CKM-based clustering consistently outperforms conventional representations, including GEM, NDM, CNDM, and kScReNI, in terms of accuracy, robustness, and biological interpretability. We further show that CSCN-derived representations preserve developmental structure in both simulated branching trajectories and human embryo data, and extend naturally to paired multiome, CITE-seq, and spatial transcriptomic settings. In a paired PBMC multiome benchmark, integrating TF-target priors and chromatin accessibility improves both clustering and regulatory-edge precision relative to existing methods. Controlled confounding simulations further show that CSCN consistently achieves the lowest false-positive rates compared with CSN and c-CSN. Additionally, CSCN facilitates biomarker discovery by identifying causal genes directly linked to disease phenotypes, thereby providing mechanistic insight into disease processes.

In summary, CSCN offers the first scalable framework for inferring directed, cell-specific gene regulatory networks, from which CKM is derived to support accurate, interpretable, and robust downstream analyses.

## 2 Methods

### 2.1 Construction of CSCN

**Figure 1:**
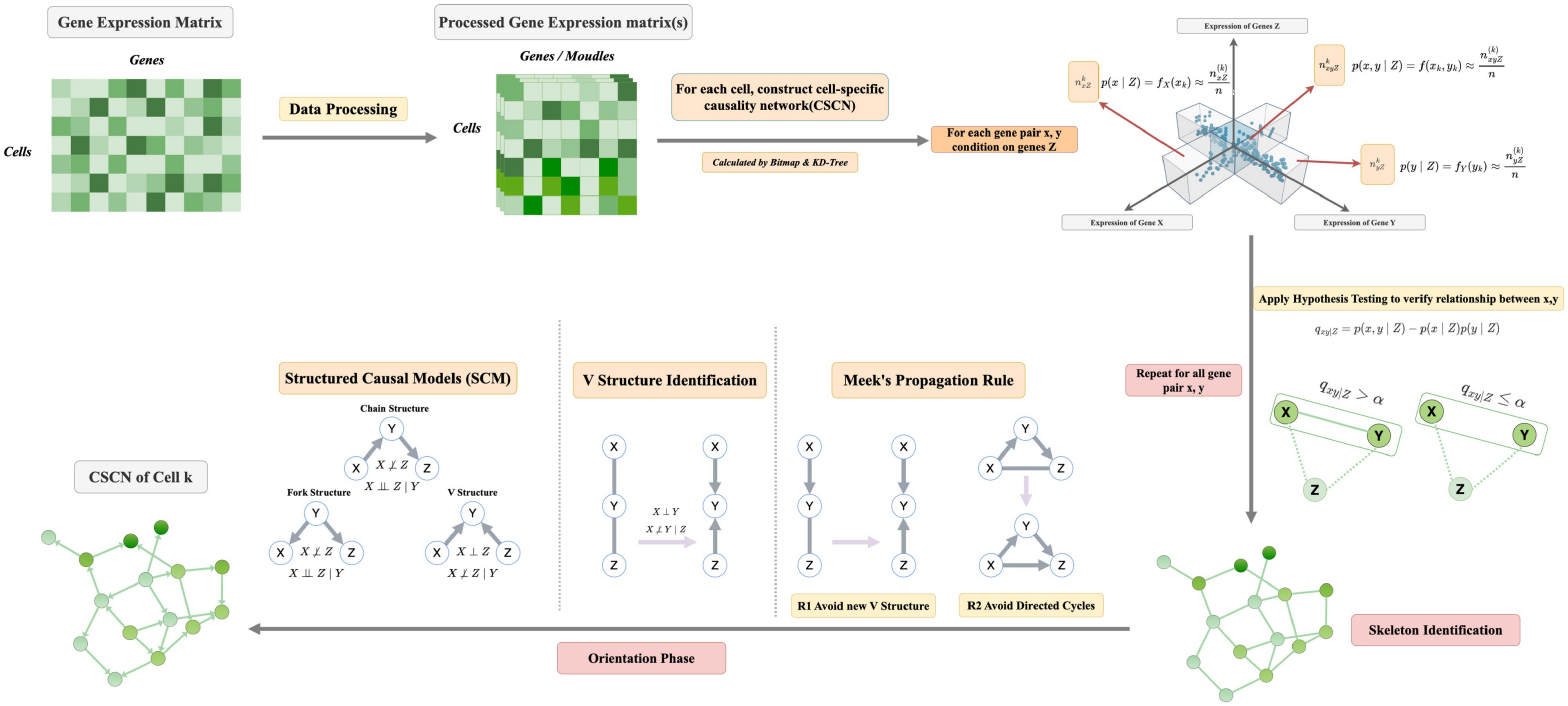
Workflow of CSCN. Starting from single-cell expression matrices, preprocessing and dimensionality reduction (via NMF, WGCNA, or targeted subsets; see Supplementary Section S1.1, available as supplementary data at *Bioinformatics* online) are applied. For each cell, conditional independence testing with full conditioning sets yields a pruned undirected skeleton. The orientation phase, implemented by the Peter-Clark algorithm, assigns causal directions to form a PDAG. CSCN employs KD-tree and bitmap-based accelerations (see Supplementary Section S1.2, available as supplementary data at *Bioinformatics* online) to make full conditioning feasible, resulting in directed cell-specific causal networks.

We propose the CSCN framework to infer directed regulatory interactions from single-cell scRNA-seq data. The CSN method infers cell-specific gene networks by testing pairwise dependencies between genes, but it may overestimate network connectivity due to indirect effects. To address this issue, CSCN refines the dependence testing between two genes, A and B, by conditioning on additional genes. Unlike c-CSN, which conditions only on a single selected gene, CSCN considers a broader set of candidate genes, up to full conditioning on all other genes. This broader conditioning helps to better eliminate indirect dependencies in high-dimensional settings. The framework proceeds in two main steps (Fig. 1) First, dependence testing is applied to identify the network skeleton for each cell. Since using a large conditioning set greatly increases computational demands, CSCN incorporates algorithmic optimizations to improve efficiency and scalability. Second, to ensure that the resulting graph gives the causality direction, CSCN applies a causal orientation step based on the PC algorithm with Meek’s orientation rules.

#### 2.1.1 Skeleton construction

Given *m* genes (or gene modules) and *n* single cells, CSCN produces *n* partially directed acyclic graphs (PDAGs) for causality network for every cells, denoted by $\left\{G^{\left(1\right)},\ldots ,G^{\left(n\right)}\right\}$. First, CSCN incorporates a preprocessing step to reduce dimension and remove noise in scRNA-seq data (see Supplementary Section S1.1, available as supplementary data at *Bioinformatics* online). Then, for each cell k∈{1,…,n}, when we consider the interaction between two genes *x, y*, we define their conditioning gene set as $Z=\{z_{1},\ldots ,z_{r}\}$. The conditioning gene set contains the candidate gene *z* that may have x→z and z→y transfer interaction and influence the inference of the indirect edge between x→y. Then we define the localized neighborhoods for each gene in the expression space. For cell *k*, the neighborhood of a gene is an interval of predefined size centered around the expression value of that gene in cell *k*. Using these neighborhoods, we count neighboring cells $k^{\prime }$ that satisfy different co-occurrence patterns: $n_{xyZ}^{\left(k\right)}$ cells that simultaneously fall into the local neighborhood of *x*, *y* and Z, $n_{xZ}^{\left(k\right)}$ cells that fall into the local neighborhood of *x* and Z, $n_{yZ}^{\left(k\right)}$ cells that fall into the local neighborhood of *y* and Z, $n_{Z}^{\left(k\right)}$ cells that fall into the neighborhood of Z. These counts are used to construct a localized deviation statistic, which measures the strength of the conditional association between x and y given Z:


$${\hat{q}}_{xy|Z}^{\left(k\right)}=\frac{n_{xyZ}^{\left(k\right)}}{n_{Z}^{\left(k\right)}}-\frac{n_{xZ}^{\left(k\right)}\cdot n_{yZ}^{\left(k\right)}}{{\left(n_{Z}^{\left(k\right)}\right)}^{2}}.$$

We then estimate the standard normal distribution of the count and test the *P*value that a direct association exists between *x* and *y*. Under the null hypothesis x⊥y∣Z, meaning there is no direct causal relationship between x and y after conditioning on Z, the normalized statistic ${\hat{q}}_{xy|Z}^{\left(k\right)}$ approximately follows a standard normal distribution. Edges are retained if the test statistic exceeds a predefined threshold: ${\hat{q}}_{xy|Z}^{\left(k\right)}>z_{1-\alpha }$, where $z_{1-\alpha }$ is the (1−α) quantile of N(0,1). By iterating over conditioning sets of increasing size, CSCN progressively removes indirect connections, resulting in a pruned undirected network skeleton for each cell k. This refined skeleton forms the basis for the subsequent causal orientation step, ultimately producing a cell-specific PDAG.

#### 2.1.2 Orientation phase

Given the undirected skeleton, CSCN proceeds to orient edges by adapting the PC algorithm with Meek’s orientation rules. The orientation process consists of two main steps. First, v-structures are identified as follows. For every unclosed triangle (x−z−y) with *x* and *y* nonadjacent, if z∉Sep(x,y), the triangle is oriented as a collider x→z←y. Sep(x,y) denotes the set of variables that rendered *x* and *y* conditionally independent during skeleton construction. This step ensures that minimal conditional dependencies detected during skeleton construction are faithfully encoded as converging causal arrows. After v-structures are identified, additional edge directions are inferred by iteratively applying Meek’s orientation rules under the constraint of acyclicity. In particular: (R1) if a→b and b−c with *a* and *c* nonadjacent, then orient b−c as b→c to avoid introducing fake colliders; (R2) if a−c→b and a−b is undirected, then orient a−b as a→b to maintain acyclicity; (R3) if two nonadjacent vertices *c* and *d* are both oriented as c→b and d→b, while a−b, a−c, and a−d are undirected, then a−b is oriented as a→b to preserve consistency. These rules are applied repeatedly until no further orientations can be made, yielding a maximally oriented graph for each cell. Edges that remain undirected after this process correspond to relationships whose direction cannot be uniquely determined from observational data alone. The resulting graph is a PDAG $G^{\left(k\right)}$ per cell, capturing both the reliably inferred causal arrows and the undirected edges whose orientation cannot be resolved only on given data.

#### 2.1.3 Computational acceleration

Different with c-CSN that restrict Z to one conditional gene to reduce computational burden (Li *et al*. 2021), CSCN explores larger conditioning sets, up to full conditioning on all other genes. This allows CSCN to more effectively eliminate indirect associations, but it also greatly increases computational demands. Naïve exhaustive conditional testing across *m* genes and *n* cells requires on the order of $O(m^{3}2^{m}n)$ operations, as all possible conditioning sets must be enumerated. Such complexity is computationally prohibitive for large-scale single-cell datasets. To address this challenge, CSCN introduces several algorithmic optimizations to accelerate localized neighborhood queries and conditional probability estimation: First, gene expression profiles are indexed using a KD-tree, enabling neighborhood range queries to be performed in expected sublinear time $O(n^{1-\frac{1}{m}})$per query, rather than the naive linear O(n) cost. This significantly reduces the computational load in high-dimensional space. Second, conditional neighborhoods are cached using compact bitmap representations, allowing k-way set intersections to be executed via bit-parallel machine-word operations. This provides up to a 64 times speedup in practice when estimating conditional probabilities. Together, these optimizations (see Supplementary Section S1.2, available as supplementary data at *Bioinformatics* online) yield order-of-magnitude performance improvements, making full conditioning across all genes computationally feasible. This enables CSCN to construct cell-specific causal networks while accounting for the complete set of genes, thereby improving the accuracy of inferred causal structures.

### 2.2 Causal Katz matrix from CSCN

To quantify the causal influence of each gene, capturing both direct effects and indirect effects propagated through downstream regulatory pathways, we compute the Katz centrality for each cell’s CSCN. Katz centrality is a network measure that extends simple connectivity by accounting for all possible directed paths, while exponentially damping the contribution of longer paths. This makes it well-suited for modeling how perturbations to a gene may propagate through the regulatory network. For each cell k, the Katz centrality vector is defined as:


$$v^{\left(k\right)}={\left(I-\alpha A^{\left(k\right)}\right)}^{-1}\beta^{\left(k\right)}.$$

Where $A^{\left(k\right)}\in R^{m\times m}$ is the directed adjacency matrix of the inferred CSCN for cell $\beta^{\left(k\right)}\in R^{m}$ is a bias vector derived from the gene expression profile of that cell. The parameter α=0.05 is a damping factor chosen to satisfy $\alpha <1/\lambda_{\max}(A^{\left(k\right)})$, ensuring convergence of the inverse and controlling how strongly indirect effects are weighted relative to direct effects.

By concatenating the Katz centrality vectors across all cells, we construct the Causal Katz Matrix (CKM):


$$\text{CKM}={\left(v_{ik}\right)}_{i=1,\ldots ,m;\;\;k=1,\ldots ,n}$$

Where each entry $v_{ik}$ represents the causal propagation capacity of gene *i* in cell *k*. The CKM thus provides a cell-by-gene representation of causal influence, which can be directly integrated into downstream single-cell analyses such as clustering, trajectory inference, or identification of key regulatory drivers.

### 2.3 Extensions beyond scRNA-seq

CSCN can be extended beyond scRNA-seq to single-cell proteomic data, spatial transcriptomic data, and integrated multi-omics data while keeping the core skeleton construction and causal orientation framework unchanged. For single-cell proteomic data, protein abundance features such as ADT measurements can be used to infer cell-specific causal relationships among measured proteins. For spatial transcriptomic data, spatial location information can be incorporated to construct spatially informed representations before CSCN inference, enabling causal network reconstruction in a spatial context. For multi-omics data with chromatin accessibility, ATAC-derived features or TF-target prior information can be introduced during skeleton construction to guide regulatory interaction inference (More details in Supplementary Section, available as supplementary data at *Bioinformatics* online).

### 2.4 Evaluation by clustering

Following the methodologies in CSN (Dai *et al.* 2019), c-CSN (Li *et al*. 2021) and kScReNI (Xu *et al*. 2025), we evaluated the quality of the inferred networks through functional clustering performance.

Clustering based on inferred network-derived features has previously been used to identify stable cell states (Aibar *et al*. 2017, Bravo González-Blas *et al*. 2023) and to serve as a robust indicator of the biological accuracy of reconstructed networks (Xu *et al*. 2025).

In this study, we assessed the clustering performance of CSCN using nine publicly available single-cell RNA-seq (scRNA-seq) datasets (Camp *et al*. 2015, Segerstolpe *et al*. 2016, Tasic *et al*. 2016, Close *et al*. 2017, Haber *et al*. 2017, Fujimoto *et al*. 2020, Ranzoni *et al*. 2021, Leary *et al*. 2022).

Clustering was performed using five different types of input features: GEM (Gene Expression Matrix): the raw gene expression profiles. NDM (Network Degree Matrix): computed from the Cell-Specific Network (CSN). CNDM (Conditional Network Degree Matrix): derived from the conditional Cell-Specific Network (c-CSN). kScReNI: the network-derived representation generated by the k-nearest-neighbor ScReNI pipeline from scRNA-seq data alone. CKM (Causal Katz Matrix), proposed in this study, represents each cell based on its inferred causal graph. By comparing CKM-based clustering against expression-based and non-causal network-based results, we aimed to determine whether the inferred causal hierarchies provide a more biologically grounded representation of cellular identity.

Two widely used unsupervised clustering algorithms, K-means and K-medoids, were applied to each feature type. The number of clusters was set to match the known number of cell types in each dataset. Clustering accuracy was measured by computing the Adjusted Rand Index (ARI). In addition, we compared runtime performance to evaluate the computational efficiency of CSCN relative to CSN and c-CSN. For visualization, UMAP (McInnes *et al*. 2018) and t-SNE were applied to the feature matrices for dimensionality reduction. The resulting embeddings were plotted to qualitatively compare cluster separability across feature representations.

### 2.5 Biomarker discovery

We applied CSCN to integrate breast cancer single-cell data (Xu *et al*. 2024) with causal effect estimation to identify genes that have causal influences on diseases (Supplementary Fig. S3. Biomarker Discovery, available as supplementary data at *Bioinformatics* online). Gene expression profiles were obtained from cancer and healthy control samples, and CSCNs were constructed for each cell. These networks were then merged into a global causal network by including any edge present in at least one cell’s CSCN, ensuring broad coverage of potential causal interactions across diverse cell states.

To link genes to disease status, we added a virtual disease node, assigned a value of 1 for cancer cells and 0 for healthy cells. The goal is to estimate the Average Causal Effect (ACE) (Pearl 2009, Pearl and Mackenzie 2018) of each candidate gene *T* on disease outcome *Y*, while adjusting for confounders *C*, factors that influence both *T* and *Y* and can produce spurious associations.

Using Pearl’s backdoor adjustment formula (Pearl 2009, Pearl and Mackenzie 2018), we can estimate the expected disease outcome under a hypothetical intervention on the candidate gene:


$$E[Y|\text{do}(T=t)]=\sum_{C}E[Y|T=t,C=c]\cdot P(C=c),$$

Where *t* represents a specific expression level of *T*. Here, E[Y∣T=t,C=c] is the conditional expectation of disease status given the gene expression and confounders, and P(C=c) is the probability distribution of the confounders. We approximate E[Y∣T=t,C=c] nonparametrically using K-Nearest Neighbors (KNN):


$$E[Y|T=t,C=c]\approx \frac{1}{K}\sum_{j\in K-\text{nearest}\;\text{neighbors}}Y_{j}$$

The ACE is then computed as the difference between interventional states:


$$\mathrm{ACE}=E[Y|do(T=t_{1})]-E[Y|do(T=t_{0})].$$

The terms $t_{0}$ and $t_{1}$ represent the interventional states of the gene’s expression, such as baseline versus elevated levels, allowing us to simulate the effect of altering the gene’s activity.

A non-zero ACE indicates that perturbing the candidate gene’s expression directly alters the probability of the cell being in a disease state, even after adjusting for confounding factors. Conversely, an ACE near zero suggests that any association between the gene and disease status is likely non-causal or explained by other variables. Thus, genes with non-zero ACE values are considered causal biomarkers, as they play a direct and potentially actionable role in disease mechanisms rather than merely exhibiting correlative patterns.

## 3 Result

### 3.1 Evaluation by clustering

To assess the quality of the inferred causal networks, we performed clustering analyses using the CKM representation across nine publicly available single-cell RNA-seq (scRNA-seq) datasets (Camp *et al*. 2015, Segerstolpe *et al*. 2016, Tasic *et al*. 2016, Close *et al*. 2017, Haber *et al*. 2017, Fujimoto *et al*. 2020, Ranzoni *et al*. 2021, Leary *et al*. 2022).

CKM is derived from the CSCN by computing Katz centrality for each gene, which captures both direct regulatory effects and multi-step causal propagation. Using the K-means algorithm, CKM achieved the highest adjusted Rand index (ARI) on 5 out of 9 datasets (55.6%). With K-medoids, CKM ranks first on 8 out of 9 datasets (88.9%). Overall, across all 18 algorithm–dataset combinations, CKM achieved the top ARI in 13 cases (72.2%) (Table S1, available as supplementary data at *Bioinformatics* online).

**Figure 2:**
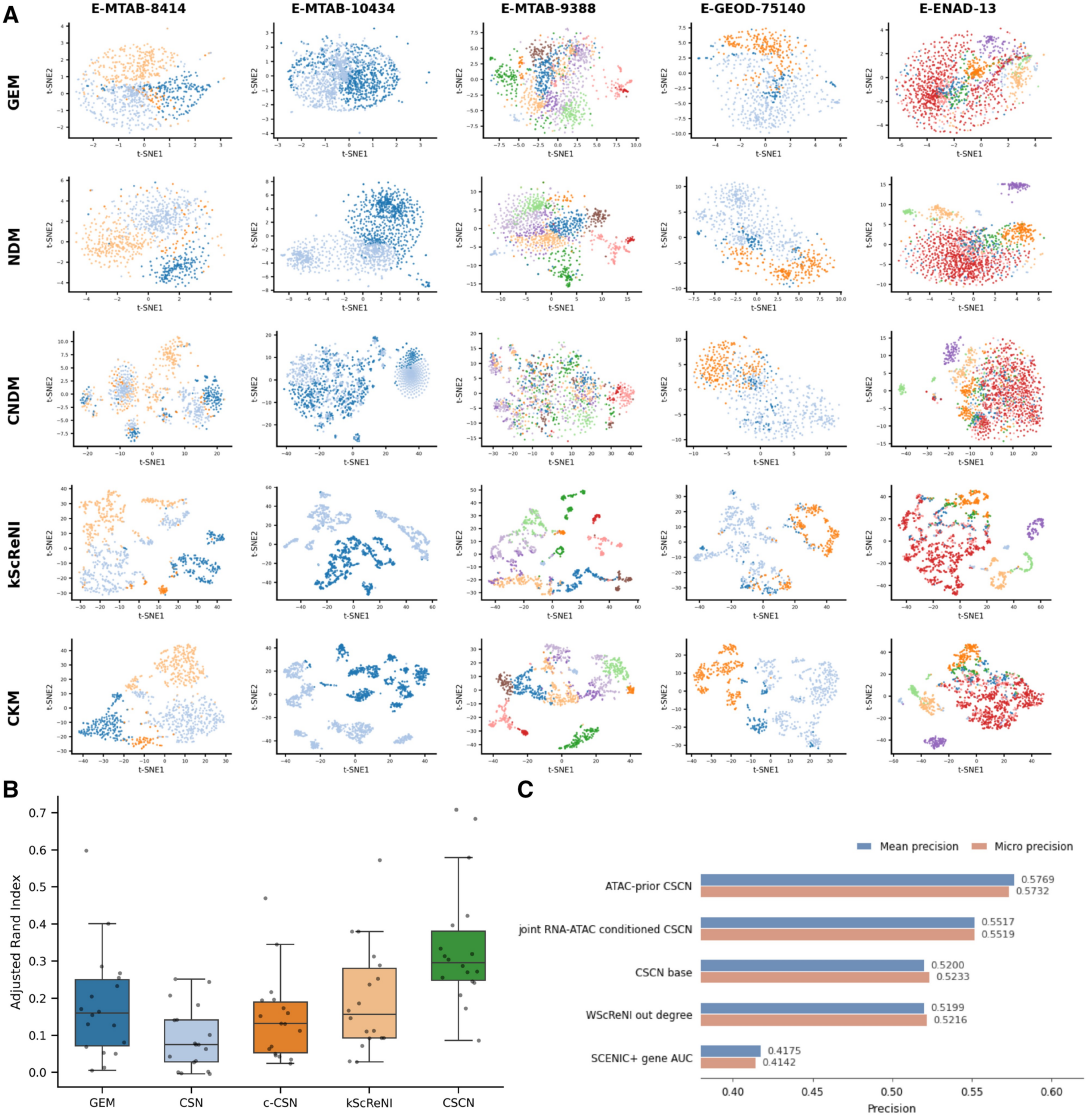
Regulatory precision and clustering performance comparison across feature representations. (A) t-SNE visualizations of clustering structure. (B) ARI distributions across methods. (C) Regulatory precision of CSCN, ScReNI, and SCENIC+.

To assess feature space separability, we visualized the embeddings using t-SNE (Fig. 2A). CKM-based embeddings displayed clearer cluster boundaries, with reduced overlap between clusters and tighter within-cluster grouping. By contrast, representations from GEM, NDM, and CNDM showed greater inter-cluster mixing. kScReNI produced more structured separation in several datasets, but its cluster boundaries remained less distinct than those of CKM overall, consistent with the ARI summaries in Fig. 2B. Aggregate ARI statistics are shown in boxplots (Fig. 2B). CKM not only achieved the highest median ARI but also exhibited the smallest interquartile range (IQR), indicating both better performance and greater stability across datasets. Furthermore, we verified the structural integrity of the inferred networks by analyzing the size of the Largest Connected Component (LCC). Across all datasets, the LCC ratios consistently exceeded the non-trivial connectivity threshold of 0.1, with medians ranging from 0.15 to 0.25 (see Supplementary Section S2.1 and Supplementary Fig. S4 & 4, available as supplementary data at *Bioinformatics* online for detailed LCC size distributions). These results confirm that while CSCN utilizes conservative significance thresholds to minimize false positives, it maintains a stable connected regulatory core compatible with biological plausibility.

### 3.2 Validation of CSCN across multi-modal and temporal settings

We evaluated whether CSCN-derived representations preserve temporal structure and generalize beyond scRNA-seq. In dyngen bifurcating and trifurcating simulations, CSCN(NMF) CKM achieved the highest trajectory scores across both kNN- and MST-based graphs (0.7283 and 0.7435 for bifurcation; 0.7395 and 0.7396 for trifurcation). In a human embryo dataset spanning embryonic day 3 to day 7, CSCN(NMF) showed the strongest agreement with developmental progression, achieving the highest Spearman correlation (0.8773), pairwise ordering accuracy (0.9363), and terminal day 7 enrichment (0.8760). These results indicate that CSCN preserves both lineage separation and continuous temporal progression (Supplementary Section S2.2, available as supplementary data at *Bioinformatics* online).

We further tested CSCN in chromatin-informed, protein-level, and spatially resolved settings. In the PBMC multiome benchmark, ATAC-prior CSCN improved clustering ARI from 0.5617 for RNA-only CSCN to 0.6158, outperforming SCENIC+ gene AUC (0.4397) and wScReNI outdegree (0.6017). In spatial transcriptomics data from SCP2046 c1, CKM ARI increased from 0.4342 in the base model to 0.5449 after using spatial information (Supplementary Sections S2.3–S2.5, available as supplementary data at *Bioinformatics* online). In the PBMC CITE-seq dataset GSE128639, applying CSCN to protein ADT features preserved the advantage of protein-level measurements (CKM ADT: ARI = 0.6384, NMI = 0.7478), while joint RNA+ADT CKM achieved the best performance (ARI = 0.6805, NMI = 0.7694).

Finally, we assessed whether CSCN recovers biologically plausible regulatory edges in the PBMC multiome setting using ChIP-Atlas immune TF-target support as an external reference. RNA-only CSCN achieved mean and micro precision of 0.5200 and 0.5233, comparable to wScReNI outdegree and higher than SCENIC+ gene AUC. Adding chromatin-derived regulatory priors further improved precision, with ATAC-prior CSCN reaching the highest mean and micro precision (0.5769 and 0.5732) (Fig. 2C) (Supplementary Sections S2.6, available as supplementary data at *Bioinformatics* online). We also conducted controlled simulation experiments to assess the impact of confounding regulation; CSCN consistently achieved the lowest false-positive rate across all settings, outperforming both CSN and c-CSN (Supplementary Section S2.7, available as supplementary data at *Bioinformatics* online). These results suggest that CSCN can incorporate auxiliary modalities without changing its core causal framework, and that chromatin accessibility is most effective when used as structured regulatory prior information during skeleton learning.

### 3.3 Temporal dynamics on network

Using CSCN, we analyzed temporal network dynamics during the 125-day differentiation of H1 human embryonic stem cells into ventral neuronal lineages (Close *et al*. 2017). To establish a functional roadmap, we first identified three key gene modules via WGCNA: the magenta module (cell cycle), turquoise (cell migration and structural remodeling), and brown (synaptic maturation) (Supplementary Fig. S7, available as supplementary data at *Bioinformatics* online). While global activity scores indicated a sequential transition from proliferation to maturation (Supplementary Fig. S7, available as supplementary data at *Bioinformatics* online), these bulk-level trends often mask the intricate heterogeneity of regulatory logic across distinct cell lineages.

**Figure 3:**
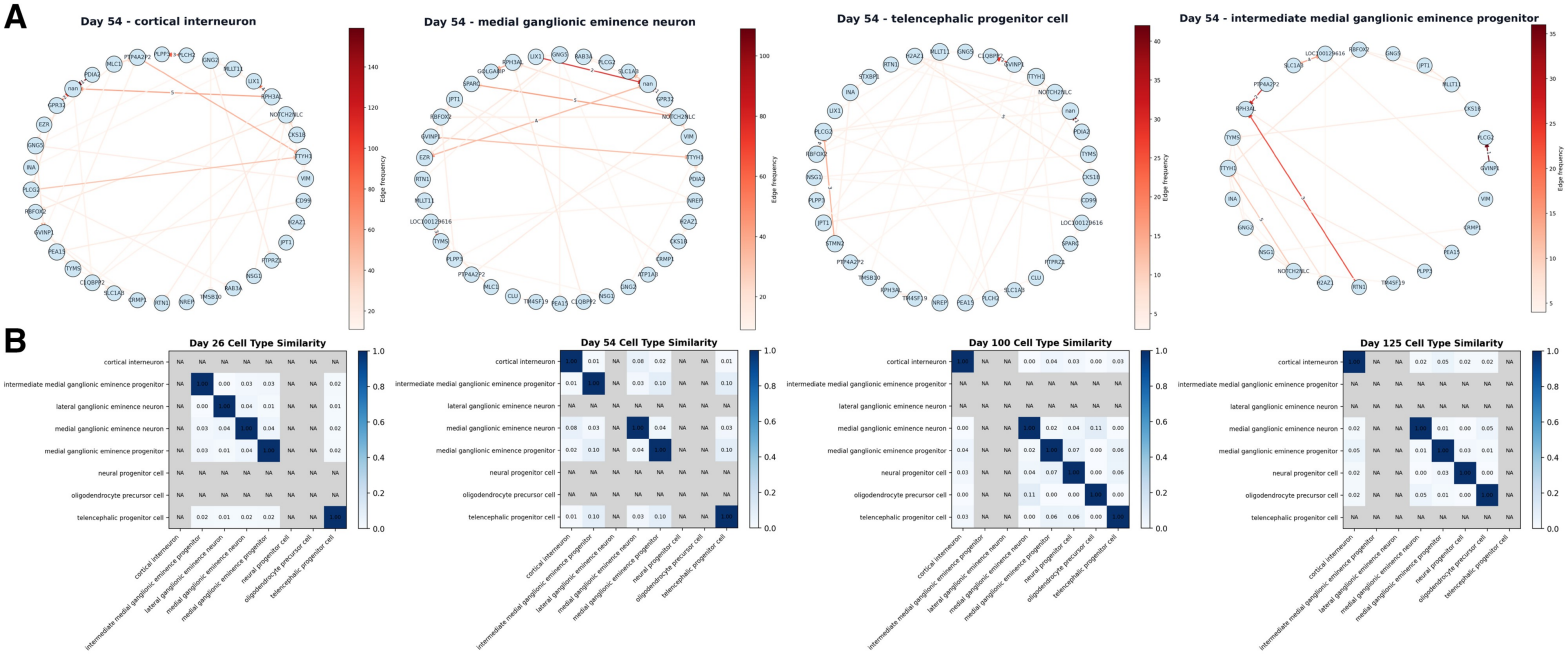
Lineage-specific consensus networks and systematic regulatory dynamics. (A) Consensus CSCNs for the turquoise module across key lineages at Day 54. Networks represent the stable regulatory backbone aggregated from hundreds of cells. (B) Jaccard similarity heatmap of consensus edge sets at Day 26, Day 54, Day 100 and Day 125, demonstrating minimal structural overlap between distinct cell types.

To ensure the representativeness of our findings and address the inherent stochasticity of single-cell data, we transitioned from individual cell analysis to a consensus CSCN framework. We constructed consensus CSCNs by aggregating hundreds of cell-level DAGs within each cell type × stage (e.g. N=214 for interneurons, N=166 for MGE neurons) and retaining only stable edges that recur above a statistical threshold (Fig. 3A). This approach emphasizes the reproducible regulatory backbone of the population while neutralizing technical noise.

Our refined analysis reveals that regulatory hierarchies are profoundly lineage-specific, even within shared functional modules. Quantitative evaluation using Jaccard similarity of edge sets (Fig. 3B) consistently showed minimal overlap between distinct cell types at the same developmental stage (median Jaccard index = 0.036 at Day 54). For instance, the telencephalic progenitor network exhibited a significantly higher edge density (47 edges) compared to cortical interneurons (43 edges) or MGE neurons (42 edges), indicating that each lineage employs a unique causal logic to execute developmental programs.

The temporal evolution of these consensus networks pinpointed specific causal interactions driving maturation. In the MGE neuron lineage, the network underwent a systematic “contraction” (decreasing from 42 to 11 edges), where early-stage hubs were replaced by specialized mature links. For example, in the turquoise module, the GLI3 → PHYH and SGK3 → TRIM9 interactions were identified as high-confidence consensus edges driving late-stage structural remodeling. In the brown module, interactions such as CLCN4 → DCX robustly reflected the stabilization of axonal guidance. Conversely, MGE progenitors showed a late-stage network expansion (up to 57 edges by Day 125), coinciding with peak differentiation complexity (Supplementary Fig. S8A, available as supplementary data at *Bioinformatics* online).

Finally, we validated the functional importance of edge directionality by analyzing “sender” and “receiver” gene enrichments. In LGE neurons, we observed a clear directional flow from signal initiation (e.g. neurotransmitter transport) to downstream response (e.g. G-protein signaling complexes) (Supplementary Fig. S8B, available as supplementary data at *Bioinformatics* online). In summary, by integrating global module context with lineage-specific consensus networks, CSCN provides a unified and robust view of regulatory network rewiring during human neurogenesis.

### 3.4 Biomarker discovery results

We applied the CSCN-ACE workflow to an initial list of differentially expressed genes identified by DESeq2, comparing breast tumor samples to healthy controls. By calculating the ACE, we filtered this list to isolate genes with a direct causal influence on disease state, resulting in a high-confidence set of 52 causal biomarkers. This process produced two gene groups for downstream analysis: the 52 causal biomarkers and 46 DESeq2-exclusive genes that were statistically significant but did not meet the causal criteria.

**Figure 4:**
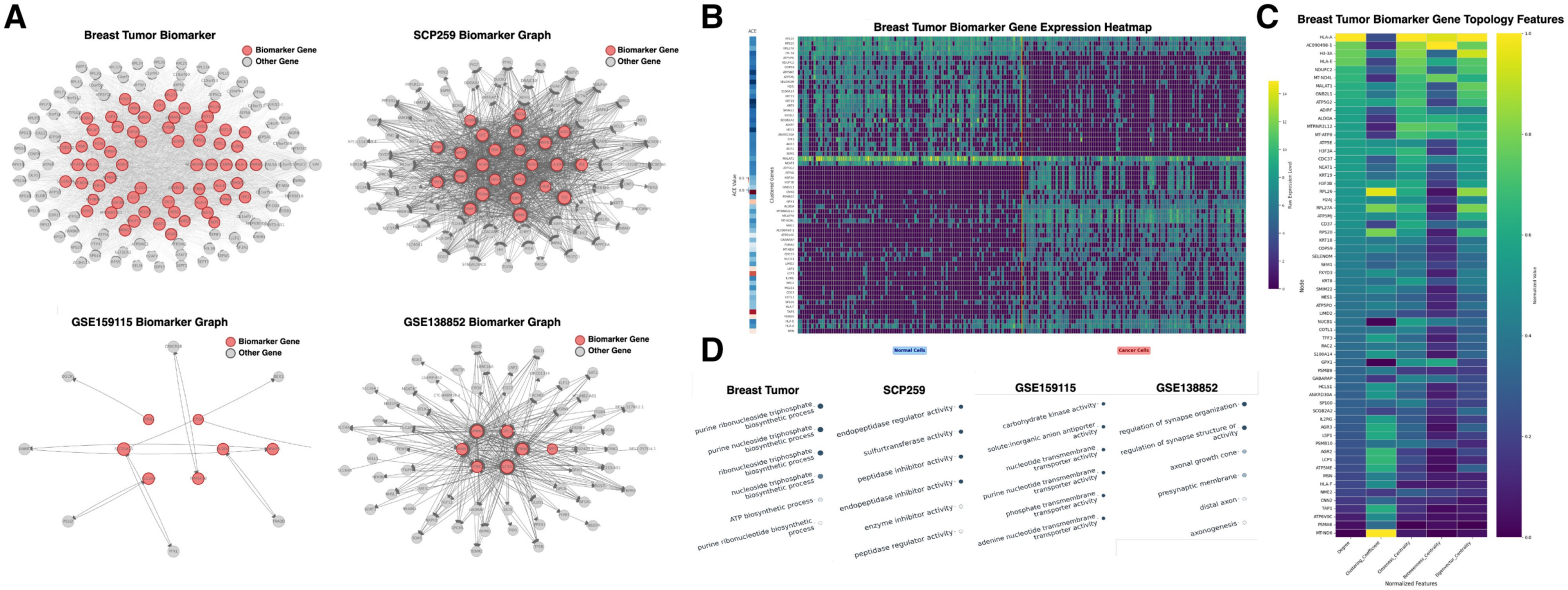
Biomarker Discovery Results. (A) Multi-disease Biomarker Causal Networks across Breast Tumor, SCP259 (Ulcerative Colitis), GSE159115 (ccRCC), and GSE138852 (Alzheimer’s Disease). Node size represents the degree of connectivity. (B) Breast Tumor Biomarker Gene Expression Heatmap. (C) Breast Tumor Biomarker Gene Topology Features Heatmap. (D) Multi-disease GO Enrichment Dotplot highlighting disease-specific functional hubs and regulatory programs across the four disease models.

Functional enrichment analysis of the 52 causal biomarkers revealed a strong convergence on two fundamental hallmarks of cancer: metabolic reprogramming and immune evasion. The KEGG pathway Oxidative phosphorylation (hsa00190, p.adjust = 9.82e-5) emerged as the most significantly enriched process. This finding was further supported by Gene Ontology (GO) analysis, which highlighted terms such as purine ribonucleoside triphosphate biosynthetic process (GO:0009206), closely linked to ATP synthesis (Fig. 4). Concurrently, the KEGG pathway ’Antigen processing and presentation’ (hsa04612) and the GO term ’antigen processing and presentation of endogenous peptide antigen via MHC class I’ (GO:0019885) were also highly enriched, suggesting that these genes play a direct causal role in shaping tumor immune recognition.

Beyond functional enrichment, these biomarkers hold topological importance within the global causal network. They frequently appear as high-connectivity hubs (Fig. 4) and exhibit elevated network feature scores (Fig. 4), reinforcing their importance. Moreover, their expression profiles show consistent and marked dysregulation between tumor and control samples (Fig. 4), reinforcing their biological relevance.

In contrast, the DESeq2-exclusive gene set was predominantly linked to basic protein synthesis. GO enrichment analysis for this group was dominated by ribosome-related processes, with cytoplasmic translation (GO:0002181, p.adjust = 9.10e-33) as the most significant term. This indicates that these genes primarily reflect the core machinery needed for rapid cell growth. Notably, strategic pathways, such as those involved in immune evasion, were entirely absent from this set. This sharp functional divergence highlights the distinction between traditional differential expression and causal inference. While DESeq2 captures the downstream effects of malignant proliferation, such as increased ribosome production, our causal method isolates the upstream regulatory drivers that actively shape the malignant phenotype.

To evaluate the generalizability of CSCN across diverse pathological contexts, we extended the framework to three additional complex disease models (Fig. 4): inflammatory mucosal disease (SCP259, Ulcerative Colitis), clear cell renal cell carcinoma (GSE159115), and Alzheimer’s Disease (GSE138852). CSCN dynamically adapted to specific disease architectures. In SCP259, it prioritized 24 biomarkers centered on mucosal barrier defense and protease inhibition (e.g. AGR2 Ye *et al*. (2021), PI3 Flach *et al*. (2006)). In the metabolic tumor setting (GSE159115), it acted conservatively to isolate 6 rate-limiting energetic bottlenecks, including the glycolytic control valve PFKL Wang *et al.* (2016). Furthermore, in the highly distributed multicellular architecture of Alzheimer’s Disease (GSE138852), it yielded a sparse panel of 7 functional hubs critical for synaptic and glial-interface integrity (e.g. SLC1A2 Lin *et al*. (2012), SPP1 Keren-Shaul *et al.* (2017)). Collectively, this multi-disease analysis confirms that CSCN consistently extracts structurally and functionally coherent biomarker panels representing true upstream pathogenic drivers across diverse complex diseases; detailed disease-specific results and interpretations are provided in Supplementary Section S2.8, available as supplementary data at *Bioinformatics* online.

## 4 Conclusion

We introduced CSCN, a scalable framework for reconstructing directed causal gene regulatory relationships for each cell while reducing false positives. By translating causal topology into informative features such as the Causal Katz Matrix, CSCN improves cell-state representation, and through measures such as ACE, it enables causal biomarker discovery. Our results further show that CSCN can be extended to paired multiome settings through TF-target priors and chromatin-informed conditioning, providing a flexible foundation for interpretable causal analysis at single-cell resolution across development and disease.

## Supplementary Material

btag480_Supplementary_Data

## Data Availability

All datasets analysed in this study are publicly available. The datasets were obtained from the EMBL–EBI Single Cell Expression Atlas under accession numbers E-ENAD-13, E-GEOD-71585, E-GEOD-75140, E-GEOD-93593, E-MTAB-10434, E-MTAB-5061, E-MTAB-8414, E-MTAB-9067, and E-MTAB-9388; from the EMBL–EBI BioStudies database under accession number E-MTAB-3929; from the Broad Institute Single Cell Portal under accession number SCP259; from the NCBI Gene Expression Omnibus under accession numbers GSE159115 and GSE138852; and from Zenodo under DOI 10.5281/zenodo.10672250.
