# Supplementary material for: CSCN: inference of cell-specific causal networks using single-cell RNA-seq data
Source: Bioinformatics. 2026 Jun 30;42(7):btag480. doi: 10.1093/bioinformatics/btag480 (PMC13387158; doi:10.1093/bioinformatics/btag480)
Supplement: btag480_Supplementary_Data [file btag480_supplementary_data.pdf]

S1. Methods

S1.1. Data Preprocessing

Depending on downstream goals and computational constraints, we apply one of three preprocessing strategies to focus and regularize causal inference:

S1.1.1. Module-constrained gene-level inference via WGCNA.

To recover full gene-level causal networks while avoiding wasted computation on biologically implausible edges, we first perform weighted gene co-expression network analysis (WGCNA) over all cells to partition genes into co-expression modules. Leveraging the sparsity of regulatory networks and the observation that regulatory interactions are enriched within co-expressed gene sets, we assume that causal edges do not exist between genes in different modules. Consequently, causal discovery is performed independently within each module, constructing gene-level subgraphs per cell. This modular constraint significantly reduces the search space by eliminating inter-module conditioning tests. To partially compensate for the representational loss induced by this assumption, we also infer a higher-level inter-module causal network by applying the CSCN procedure to module eigengenes, yielding a hierarchical (two-layer) causal representation in which module-level influences complement intra-module gene-level networks. The workflow is illustrated in Figure S1.

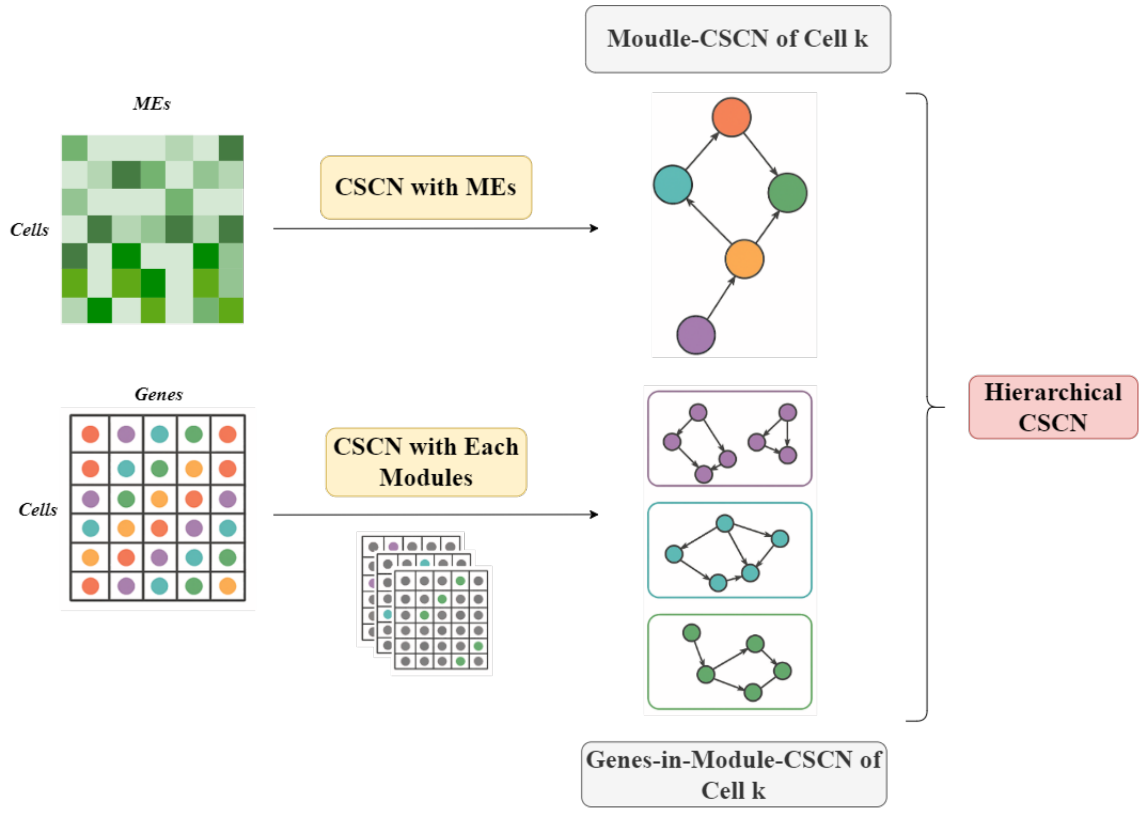

Fig. S1. Data preprocessing via WGCNA.

S1.1.2. Dimension-reduced inference via NMF.

When the objective is to transform noisy expression profiles into stable causal regulatory features rather than to recover explicit gene-level networks, we apply non-negative matrix factorization (NMF) to $X \in \mathbb{R}^{n\times m}$, $X \approx WH$ with $W \in \mathbb{R}^{n\times r}$, $H \in \mathbb{R}^{r\times m}$, $r \ll m$. Causal networks are constructed in the reduced module space of dimension r, yielding module-level CSCNs per cell; the resulting Causal Katz Matrix is then mapped back to gene resolution through the loading matrix H. The workflow is illustrated in Figure S2.

S1.1.3. Targeted subset inference.

For applications aimed at dissecting the regulatory relationships underlying specific biological processes—such as pathway-level causal intervention studies or estimation of process-specific treatment effects—we first curate a subset of genes or modules directly implicated in that process (e.g., based on pathway annotations, differential expression, or preliminary association screening) and

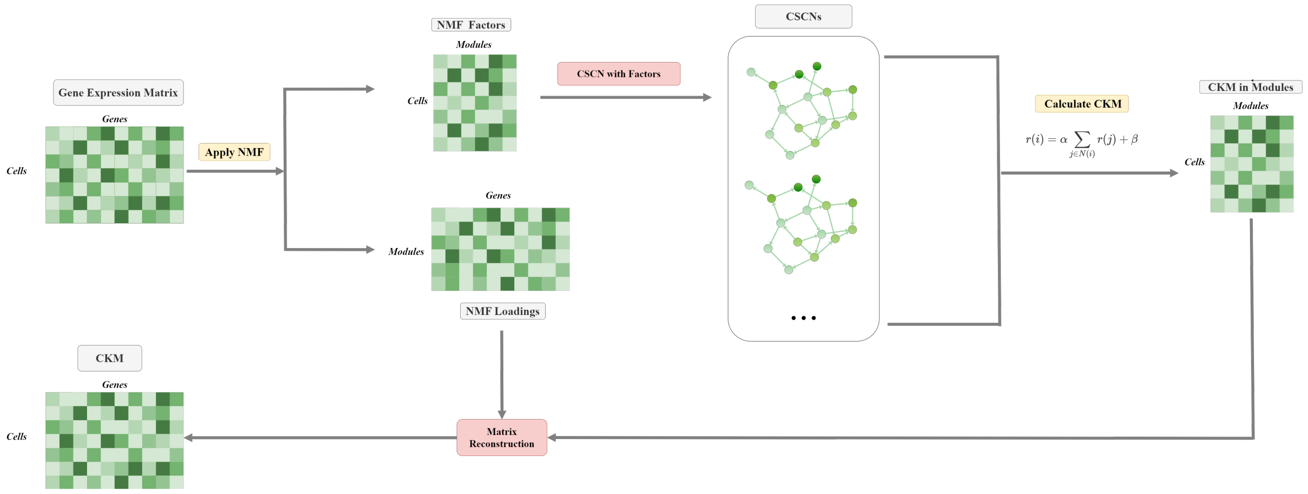

Fig. S2. Data preprocessing via NMF.

then confine the CSCN pipeline to this tailored set. This focused strategy both alleviates computational burden and filters out noise from unrelated components, thereby enabling more accurate causal recovery within the biological context of interest.

S1.2. Computational acceleration

The PC procedure is combinatorially and computationally prohibitive due to the exponential growth of conditioning sets and the cost of local neighborhood counts. To alleviate this, we first index all cell-wise expression profiles with a KD-tree, treating each cell as a point in the relevant expression subspace. Range-count queries for neighborhood construction leverage bounding-box pruning, reducing per-query cost from $O(nm)$ to approximately $O(n^{1-1/m})$ and thereby compressing the per-cell complexity to $O(m^2 2^m n^{1-1/m})$ in the full gene-level setting.

For further speedup, we layer on a bitmap-based intersection scheme with dynamic programming (DP) to efficiently compute conditional neighborhoods. Each gene (or module) and its local neighborhood is encoded as a bitset $B_g \in \{0,1\}^n$, where bit i is 1 if cell i lies in the expression interval for gene g. The joint neighborhood for a conditioning set $S = \{z_1, \dots, z_k\}$ is then the bitwise AND of its members:

$$B_S = \bigwedge_{z \in S} B_z.$$

We cache these intersections so that extensions of a known subset can be updated incrementally: for any S and a new gene $z \notin S$,

$$B_{S \cup \{z\}} = B_S \ \&\ B_z,$$

requiring only one additional bitwise AND. This DP-style transition avoids recomputing full intersections from scratch when exploring overlapping conditioning subsets. Counting cells in the intersection is then a population count on B_S, and the cost of computing/intersecting k neighborhoods reduces from $O(kn)$ to $O(k \cdot \lceil n/w \rceil)$, where w is the machine word size (e.g., 64), since each bitwise operation processes w bits simultaneously. Together, the KD-tree pruning and bitmap+DP intersection caching yield substantial practical runtime reductions, particularly under sparse conditioning regimes and overlapping subset exploration.

S1.3. Downstream Analysis

Downstream Analysis (Fig. S3): Individual CSCNs are integrated to generate a global Causality Score Matrix (CKM) and global network for population-level insights. These integrated results facilitate functional subgroup identification, biomarker discovery, pseudotime reconstruction, and entropy analysis.

S2. Results

S2.1. Largest Weakly Connected Component Analysis

To quantify the degree of fragmentation of the networks inferred across different clustering experiments, we calculated the relative size of the largest weakly connected component (LWCC) for each network:

$$r_{\text{LWCC}} = \frac{|V_{\text{LWCC}}|}{|V|} \tag{1}$$

where V denotes the full set of nodes in the corresponding network, including isolated nodes. This normalized measure facilitates comparisons among networks with different numbers of nodes across clustering experiments.

Across all clustering experiments and datasets, the median LWCC ratio ranged from approximately 0.15 to 0.25. Although the inferred networks were sparse and contained multiple disconnected components, the LWCC generally comprised a non-negligible

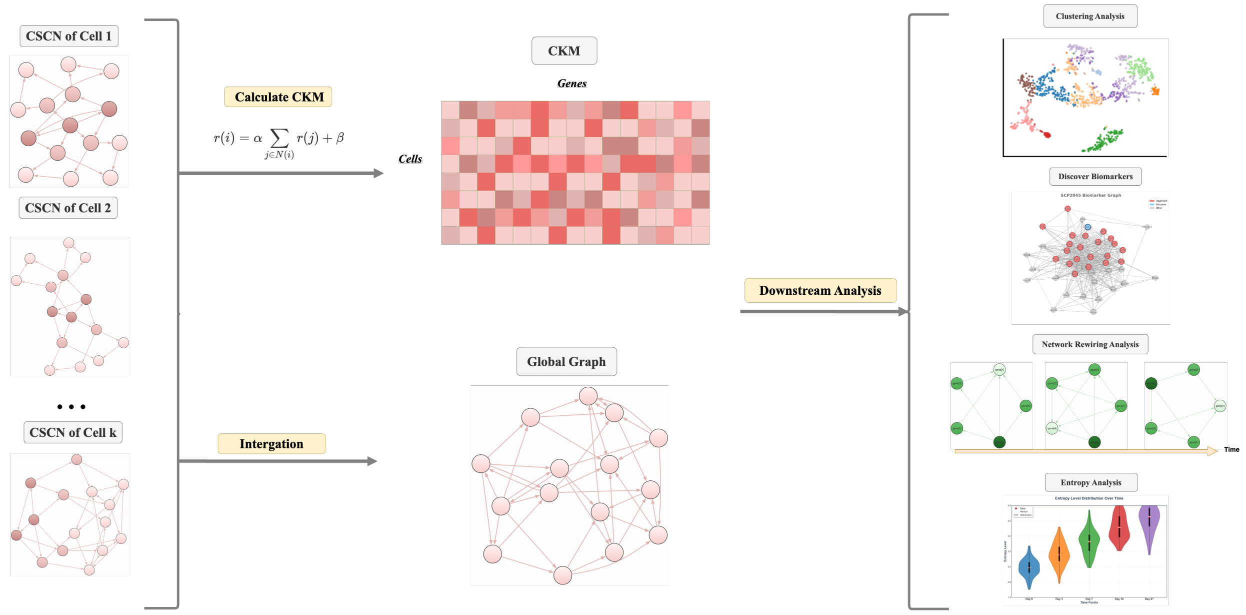

Fig. S3. Downstream Analysis of CSCN

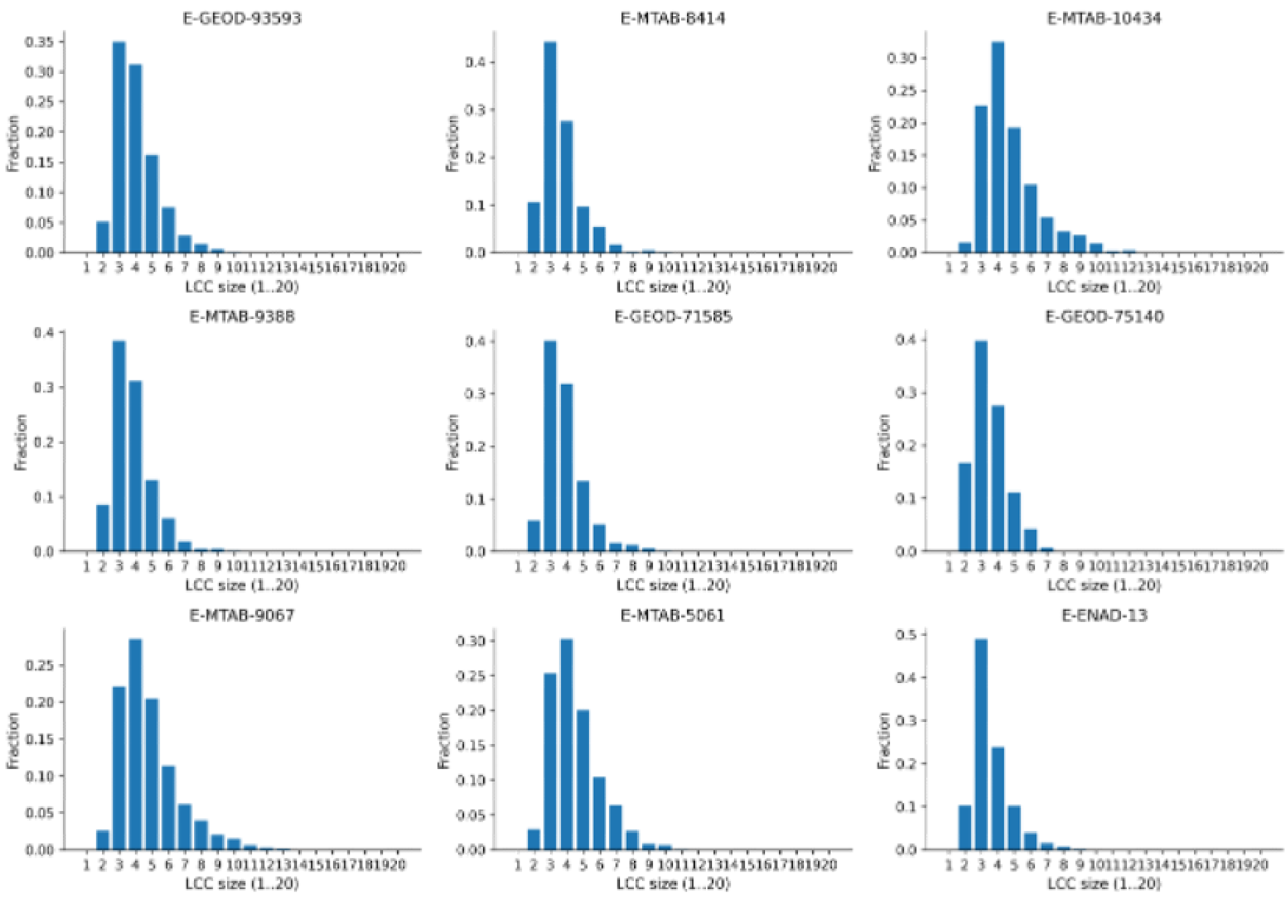

Fig. S4. Network Connectivity and Component Size Distribution

Table 1. Adjusted Rand Index (ARI) obtained with different feature inputs

Method	Input	E–GEOD 93593	E–MTAB 8414	E–MTAB 10434	E–MTAB 9388	E–GEOD 71585	E–GEOD 75140	E–MTAB 9067	E–MTAB 5061	E–ENAD 13
KMeans	GEM	0.16	0.60	0.01	0.13	0.26	0.27	0.08	**0.40**	0.13
	NDM	0.18	0.00	0.00	0.14	0.14	0.00	0.03	0.25	0.21
	CNDM	0.19	0.02	0.06	0.16	0.13	**0.47**	0.17	0.22	**0.34**
	kScReNI	0.24	0.57	0.07	0.38	0.17	0.31	**0.25**	0.38	0.29
	CKM	**0.27**	**0.68**	**0.09**	**0.42**	**0.30**	0.40	0.25	0.31	0.32
KMedoids	GEM	0.17	0.23	0.01	0.15	0.29	0.05	0.05	0.21	0.07
	NDM	0.06	0.07	0.04	0.00	0.08	0.03	0.07	**0.24**	0.10
	CNDM	0.13	0.07	0.05	0.11	0.15	0.04	0.04	0.20	0.05
	kScReNI	0.11	0.09	0.09	0.09	0.19	0.03	0.11	0.15	0.03
	CKM	**0.26**	**0.58**	**0.71**	**0.24**	**0.29**	**0.33**	**0.17**	0.22	**0.27**

Note: Boldface indicates the best result within each method–dataset pair.

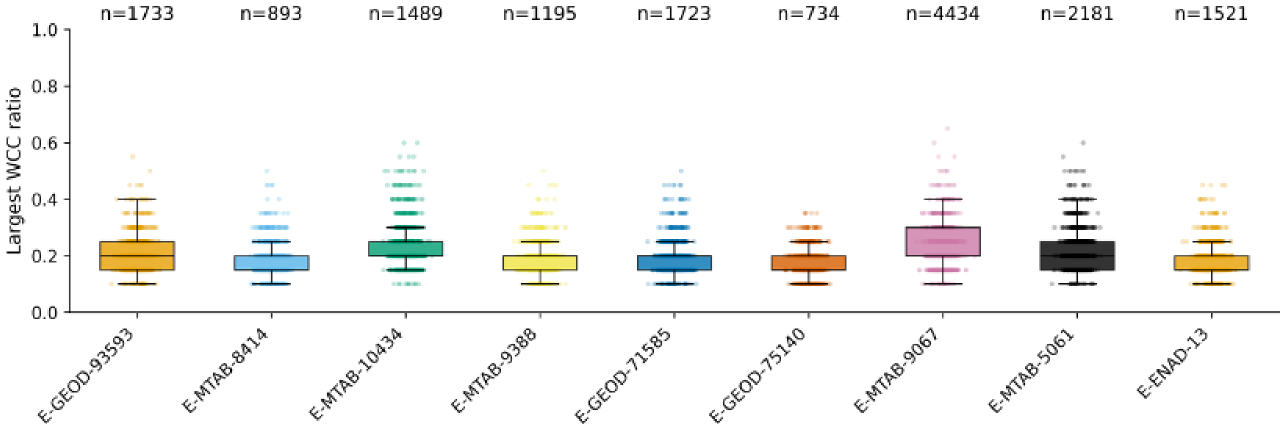

Fig. S5. Network Connectivity and Component Size Distribution

fraction of the nodes. In addition, the LWCC contained more than one node in every inferred network, indicating that none of the networks consisted entirely of isolated nodes. The distributions of component sizes and LWCC ratios across the clustering experiments are shown in Fig. S4 and Fig. S5, respectively.

The connectivity of the inferred networks is influenced by the significance level α used in conditional independence testing. Under our testing rule, an edge is removed when the corresponding conditional independence test yields a p-value greater than α. Therefore, a smaller α generally results in sparser networks and may reduce the size of the LWCC. In this study, α was selected conservatively to reduce the retention of potentially spurious edges.

S2.2. Trajectory reconstruction benchmarks

Unless otherwise specified, the same CSCN configuration was used across the trajectory and multi-omics benchmarks so that performance differences reflect the data structure and auxiliary information rather than dataset-specific retuning.

S2.2.1. Dyngen branching simulations

To evaluate CSCN under controlled developmental topologies, we generated RNA-only simulations using dyngen [Cannoodt et al., 2021]. We considered bifurcating and trifurcating trajectories, using seeds 0 and 1, with 1,000 cells and approximately 130–150 genes per simulated dataset. In total, 64 bifurcating and 72 trifurcating simulations were analyzed. For each setting, downstream trajectory structure was reconstructed on both kNN- and MST-based graphs using expression, CSCN CKM, CSCN(NMF) CKM, and kScReNI outdegree representations.

Trajectory quality was evaluated by two complementary criteria: branch assignment accuracy, quantified by branch ARI, and within-branch temporal ordering, quantified by branch-wise Spearman correlation. We combined these components into a single score using $A = \max(\text{branch ARI}, 0)$, $S = (\text{branch-wise Spearman}+1)/2$, and Score $= \sqrt{A \cdot S}$. This score penalizes failures in either branch separation or pseudotime recovery, thereby favoring representations that preserve both aspects of trajectory structure.

S2.2.2. Human embryo trajectory reconstruction

We evaluated CSCN-derived trajectory reconstruction on a human embryo dataset spanning five ordered developmental stages from embryonic day 3 to embryonic day 7. The reference trajectory is linear, progressing from embryonic day 3 to embryonic day 7.

Table 2. Trajectory reconstruction performance on dyngen branching simulations. The combined score summarizes branch assignment accuracy and branch-wise pseudotime consistency.

Graph	Topology	Method	Score
kNN	bifurcating	CSCN CKM	0.6763
kNN	bifurcating	CSCN(NMF) CKM	**0.7283**
kNN	bifurcating	Expression	0.6731
kNN	bifurcating	kScReNI outdegree	0.4177
kNN	trifurcating	CSCN CKM	0.5637
kNN	trifurcating	CSCN(NMF) CKM	**0.7395**
kNN	trifurcating	Expression	0.5437
kNN	trifurcating	kScReNI outdegree	0.5104
MST	bifurcating	CSCN CKM	0.6759
MST	bifurcating	CSCN(NMF) CKM	**0.7435**
MST	bifurcating	Expression	0.6745
MST	bifurcating	kScReNI outdegree	0.4168
MST	trifurcating	CSCN CKM	0.5758
MST	trifurcating	CSCN(NMF) CKM	**0.7396**
MST	trifurcating	Expression	0.5411
MST	trifurcating	kScReNI outdegree	0.5130

Trajectory inference was performed with PCA using 15 components, K-means clustering with $k = 3$, and Slingshot. The inferred pseudotime was evaluated against the true developmental stage labels using Spearman correlation, median stage ordering, pairwise ordering accuracy, root enrichment, and terminal enrichment.

Table 3. Trajectory reconstruction performance on the human embryo dataset.

Method	Spearman	Median order	Pairwise accuracy	Root day 3 enrichment	Terminal day 7 enrichment
GEM	0.7333	True	0.8502	0.5814	0.6202
kScReNI-OUT	0.7661	True	0.8635	0.5814	0.8217
CSCN(NMF)	**0.8773**	True	**0.9363**	0.5814	**0.8760**

CSCN achieved the best overall trajectory reconstruction performance, with the highest Spearman correlation, pairwise ordering accuracy, and terminal day 7 enrichment. All three methods preserved the correct median ordering of developmental stages, while CSCN showed the strongest agreement with the reference developmental progression.

S2.3. PBMC multi-omics analysis

To assess whether CSCN can incorporate chromatin-derived regulatory information beyond RNA expression alone, we analyzed the 10x Genomics PBMC multiome dataset, which contains paired scRNA-seq and scATAC-seq profiles measured from the same cells. Cell-type labels generated by Azimuth PBMC reference mapping were used as ground-truth labels only for ARI evaluation and were not used during CSCN network inference.

For the chromatin-informed CSCN variants, ATAC information was first converted into TF gene-target gene regulatory priors. Specifically, TF-peak evidence was integrated with ATAC-derived peak-gene links: peak-gene links were established based on positive associations between peak accessibility and gene expression, and TF binding or motif evidence was then used to connect TF genes to the corresponding peaks. This yielded chromatin-supported TF gene-target gene priors through the path TF gene–peak–target gene. Thus, the chromatin prior used by CSCN was represented at the gene-gene level rather than at the peak level.

We compared three CSCN settings. First, RNA-only CSCN used only the RNA expression matrix and served as the baseline. Second, ATAC-prior CSCN incorporated TF gene-target gene priors during both skeleton learning and edge orientation. During conditional independence testing, prior-supported TF gene-target gene pairs were protected from being prematurely removed solely by local statistical fluctuation, thereby reducing blind independence decisions for biologically supported regulatory relationships. During the PC orientation step, the same priors were used to correct edge directions that contradicted the supported TF-to-target direction. Third, joint RNA-ATAC-conditioned CSCN further incorporated chromatin accessibility into neighborhood counting. ATAC information was first mapped to the same gene-level feature space as the scRNA-seq matrix. For each gene or conditioning set, local neighborhoods were then defined by the intersection of RNA-expression neighborhoods and ATAC-derived neighborhoods, making conditional independence testing depend jointly on transcriptomic similarity and chromatin-accessibility similarity.

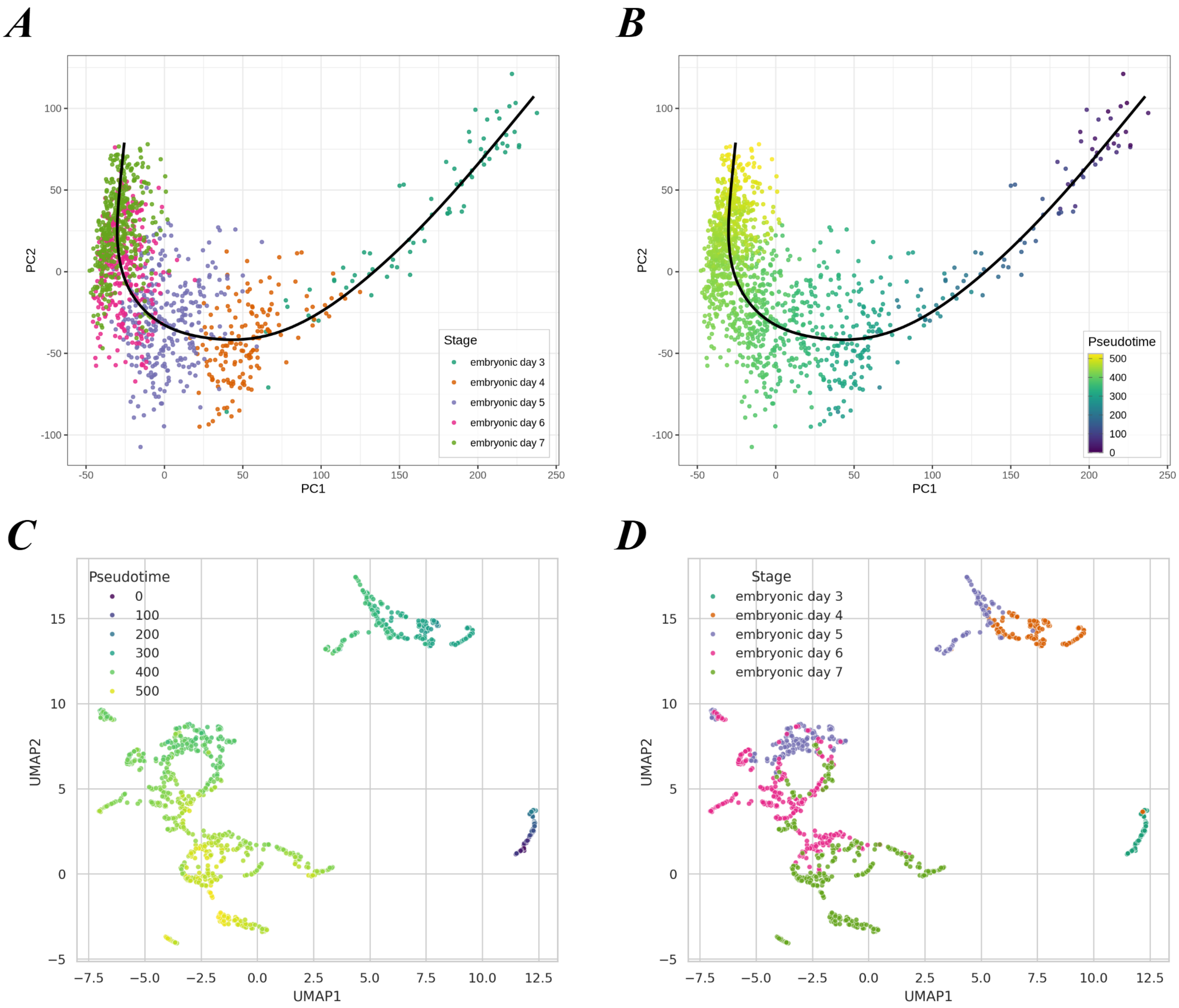

Fig. S6. CSCN-Slingshot trajectory inference on the human embryo dataset. (A) PCA colored by true developmental stage. (B) PCA colored by inferred pseudotime. (C) UMAP colored by inferred pseudotime. (D) UMAP colored by true developmental stage.

Cell-state representation benchmark.

We benchmarked CSCN-derived CKM features against ScReNI outdegree and SCENIC+ gene AUC under the same cell-state clustering evaluation. The RNA-only CSCN baseline achieved an ARI of 0.5617. Incorporating chromatin-derived regulatory priors improved clustering agreement, with ATAC-prior CSCN reaching an ARI of 0.6158, outperforming both ScReNI outdegree (0.6017) and SCENIC+ gene AUC (0.4397). Joint RNA-ATAC-conditioned CSCN achieved a similar ARI of 0.6121.

Regulatory-edge precision benchmark.

Regulatory precision was evaluated using immune-cell TF-target interactions from ChIP-Atlas as an external reference. Because different methods produced different numbers of directed edges per cell, regulatory precision was computed under a matched per-cell edge-count setting, using the same number of selected edges for each method within each cell. The RNA-only CSCN baseline achieved mean precision of 0.5200 and micro precision of 0.5233, comparable to ScReNI outdegree (0.5199 and 0.5216) and higher than SCENIC+ gene AUC (0.4175 and 0.4142). ATAC-prior CSCN achieved the best regulatory precision, increasing mean precision to 0.5769 and micro precision to 0.5732, while joint RNA-ATAC-conditioned CSCN showed intermediate precision (0.5517 and 0.5519). These results suggest that chromatin accessibility is most effective in CSCN when used as structured regulatory prior information during skeleton learning.

S2.4. Extension of CSCN to spatial transcriptomics

To leverage spatial information in ST data, we first applied local spatial smoothing to nearby spots. This step acts as a convolution-like aggregation of neighborhood signals, which helps stabilize noisy measurements and capture locally shared regulatory patterns

Table 4. PBMC multi-omics benchmark. Regulatory-edge precision was evaluated against ChIP-Atlas-supported TF-target interactions, and clustering agreement was evaluated by ARI against Azimuth PBMC cell-type labels.

Method	Mean precision	Micro precision	ARI
CSCN base	0.5200	0.5233	0.5617
ATAC-prior CSCN	**0.5769**	**0.5732**	**0.6158**
joint RNA-ATAC-conditioned CSCN	0.5517	0.5519	0.6121
ScReNI outdegree	0.5199	0.5216	0.6017
SCENIC+ gene AUC	0.4175	0.4142	0.4397

Table 5. Matched spatial-transcriptomics comparison on SCP2046 c1. All methods used the same expression input and CSCN backbone; only the use of spatial information differed.

Method	Spatial usage	CKM ARI
Base	no spatial information	0.4342
Spatial smoothing	6-NN spatial smoothing	0.5226
Spatial refinement	smoothing + self-prior refinement	0.5449

under the assumption that neighboring cells often exhibit partially similar functions. However, excessive smoothing may also wash out genuine heterogeneity within local regions. To avoid this loss of cell-specific structure, we then performed a second round of CSCN on the smoothed representation, using the stable edges from the first pass as a weak prior. This two-stage strategy allows CSCN to absorb spatial context while preserving local cellular heterogeneity, and the improved CKM ARI after refinement supports this design.

To test this idea, we analyzed the SCP2046 `c1` sample (cancer1) [Vandenbon et al., 2023] under a matched design. We restricted the analysis to the four most frequent `Category` labels (0, 1, 3, and 6), and downsampled each class to 138 spots, yielding a balanced subset of 552 spots. The same top1000 genes, module definitions, and CSCN backbone were used across all variants, so the only difference between methods was how spatial information entered the pipeline.

We compared three matched settings: Base, Spatial smoothing, and Spatial refinement. Base applied CSCN directly to the top1000-gene expression matrix. Spatial smoothing incorporated local 6-nearest-neighbor averaging before network learning. Spatial refinement further reused stable first-pass within-module edges as a weak self-prior for a second CSCN pass. Performance was assessed by CKM ARI on the balanced `Category` labels.

CKM ARI increased from 0.4342 for Base to 0.5226 for Spatial smoothing and 0.5449 for Spatial refinement (Table 5). These results support the view that spatial neighborhood aggregation improves CSCN-derived representations, while the second-pass refinement recovers local heterogeneity that could otherwise be blurred by smoothing.

S2.5. CITE-seq protein-aware extension

To evaluate whether CSCN can incorporate protein-level measurements, we analyzed the PBMC CITE-seq dataset GSE128639 [Stuart et al., 2019], which contains matched RNA and ADT profiles from the same cells. This experiment was designed to test three simple integration modes: RNA-only, ADT-only, and direct RNA+ADT concatenation. In the joint setting, RNA and ADT features were aligned by cell barcode, normalized in a modality-specific manner, and concatenated into a single expression matrix before CSCN inference.

Data preprocessing and integration.

RNA values were transformed by library-size normalization followed by `log1p`, whereas ADT values were transformed by a centered-log-ratio-like normalization. Features were then ranked by within-modality variance. For the RNA-only and ADT-only benchmarks, the top 150 RNA or ADT features were retained. For the joint benchmark, the top 100 RNA features and top 50 ADT features were selected and concatenated into a single 150-dimensional matrix. ADT features in the joint matrix were prefixed with `ADT_` to preserve modality identity during downstream analysis.

Benchmark summary.

For each representation, we compared both the raw feature matrix and the CSCN-derived CKM representation. Features were standardized before clustering, and k-means was applied with the number of clusters fixed to the 8 reference labels in the benchmark subset. Protein measurements were more informative than RNA alone: ADT outperformed RNA in both raw and CSCN-derived representations, and the joint RNA+ADT representation achieved the best overall clustering agreement after CSCN transformation. In particular, CSCN on the joint matrix improved ARI to 0.6805 and NMI to 0.7694, indicating that transcript and protein signals provide complementary information for recovering PBMC cell states.

S2.6. Simulation on confounding effects

We evaluated the proposed CSCN against the CSN and c-CSN using a controlled simulation designed to test the impact of confounding regulation. In this simulation, four genes were modeled: two independent regulators (Z_1, Z_2 and two downstream genes (X, and Y). Z_1 and Z_2 were independently sampled from distinct normal distributions and jointly regulating X and Y. Importantly, there was no direct causal link between X and Y, meaning any inferred edge between them would represent a false

Table 6. Clustering performance for GSE128639 under RNA-only, ADT-only, and joint RNA+ADT representations.

**Input modality**	**Feature type**	**ARI**	**NMI**
RNA	Raw RNA expression	0.5480	0.6644
ADT	Raw protein abundance	0.6363	0.7469
RNA+ADT	Raw joint RNA-protein	0.6255	0.7412
RNA	CKM	0.5278	0.6775
ADT	CKM	0.6384	0.7478
RNA+ADT	CKM	**0.6805**	**0.7694**

positive caused by shared regulators. We varied the regulatory strength of Z_1 and Z_2 from 0.1 to 2.0 and measured the false-positive rate (FPR) of each method. As the confounding influence increased, CSN's FPR rose sharply, starting at 0.111 and reaching 0.878. c-CSN performed better but still showed substantial confounding, with FPR increasing from 0.039 to 0.619. In contrast, CSCN maintained consistently low FPR, increasing only modestly from 0.027 to 0.148, even under the strongest confounding effects. These results demonstrate that CSCN consistently achieves the lowest false-positive rate across all parameter levels, clearly outperforming both CSN and c-CSN. This demonstrates CSCN's superior specificity and robustness for accurate gene network inference in the presence of confounding regulation.

S2.7. Temporal Dynamics on Network

Using CSCN, we analyzed temporal network dynamics with dataset E-GEOD-93593, which profiles a 125-day differentiation protocol converting H1 human embryonic stem cells into various ventrally derived cell types [Close et al., 2017]. We examined functional roles and regulatory rewiring across four developmental stages (Days 26, 54, 100, and 125).

From a functional perspective, three key gene modules involved in differentiation were identified through WGCNA analysis [Langfelder and Horvath, 2008]. At the global level, these modules displayed distinct functional enrichments (Fig. S7A): the turquoise module was enriched for cell migration, fatty acid metabolism, and extracellular matrix remodeling; the brown module was associated with neuronal development and synapse formation; and the magenta module was linked to DNA replication and cell cycle regulation. These results suggest that each module corresponds to a distinct biological program activated during stem cell differentiation.

Beyond static enrichment profiles, the functional activity of these modules shifted dynamically over time. Specifically, at a given module and time point, we calculate a score by accumulating the number of all its internal regulatory edges (i.e., the edges of the source gene to the target gene). This method allows us to plot the total regulatory activity of each module at different time points (Fig. S7B), thus revealing its dynamic functional involvement processes. Turquoise module activity intensified after Day 100, driven by interactions such as GLI3 → PHYH and SGK3 → TRIM9, reflecting late-stage structural remodeling. The brown module, linked to axon guidance and synaptic signaling, peaked at Days 100 and 125, with interactions like CLCN4 → DCX and PAK3 → CHGA becoming dominant. Meanwhile, the magenta module, associated with DNA replication and repair, surged at later stages through regulatory links such as TCF19 → TCF19 and E2F2 → E2F2. Together, these temporal dynamics suggest a sequential engagement of structural remodeling, neuronal maturation, and proliferative programs as differentiation progresses.

Our CSCN analysis uncovered that the gene regulatory networks (GRNs) of these modules are not uniform across cell types. For example, Fig. S7B illustrates how turquoise module interactions diverge between different cell populations, demonstrating distinct causal wiring despite shared module membership. This cell specificity underscores the advantage of our causal framework: it captures not only when modules are active, but also how their regulatory logic is rewired in a cell-specific manner. In summary, combining global enrichment (Fig. S7A), temporal dynamics Fig. S7B, and cell-specific causal inference Fig. S7C provides a comprehensive view of network rewiring during stem cell differentiation into neurons. In summary, CSCN integrates global enrichment, temporal dynamics, and cell-specific causal inference to provide a unified view of regulatory network dynamics.

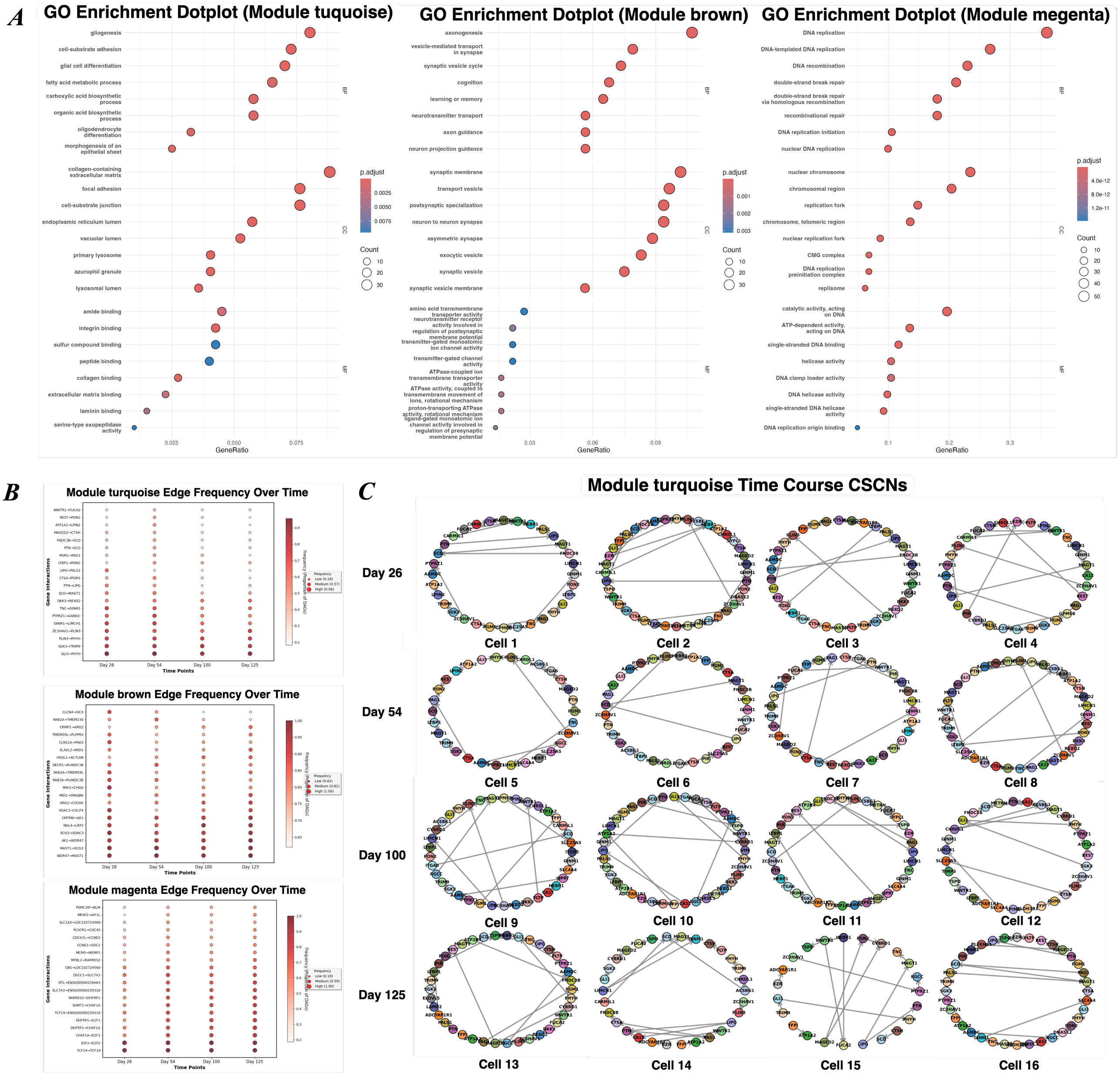

Fig. S7. Network Rewiring Results

(A) GO enrichment dotplots showing key WGCNA modules. Dot size represents gene count; color indicates statistical significance (p-value). (B) Temporal dynamics of gene interaction frequencies in WGCNA modules. (C) Time-course CSCNs for module turquoise. Rows represent different times (Day 26, Day 54, Day 100, Day 125), with a list of randomly selected cells at that time.

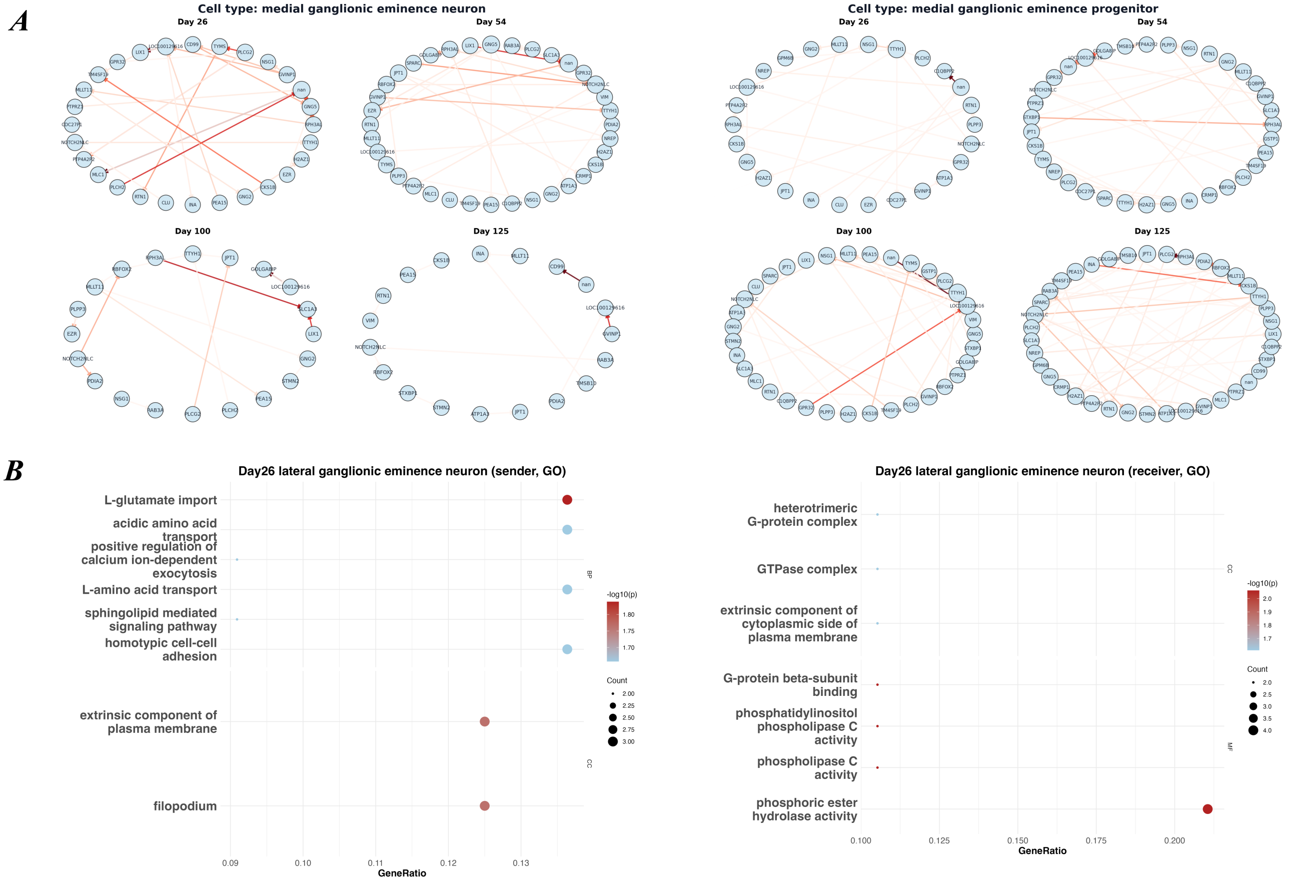

Fig. S8. Lineage-specific consensus networks and systematic regulatory dynamics. (A) Temporal trends of network complexity (edge counts) for MGE neurons and progenitors. (B) Functional asymmetry of edge directionality in Day 26 LGE neurons. Sender genes (signal sources) are enriched for neurotransmitter transport, while receiver genes (targets) are linked to downstream signaling complexes.

S2.8. Cross-dataset biomarker validation

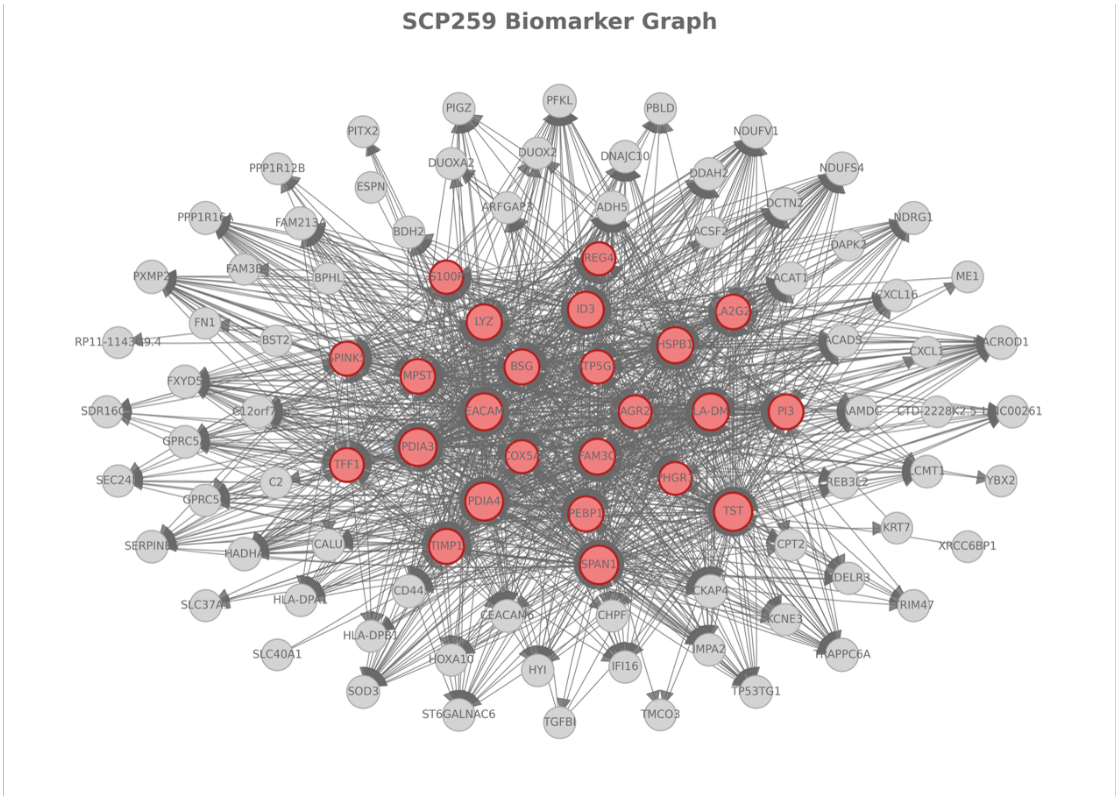

Fig. S10. SCP259 biomarker network

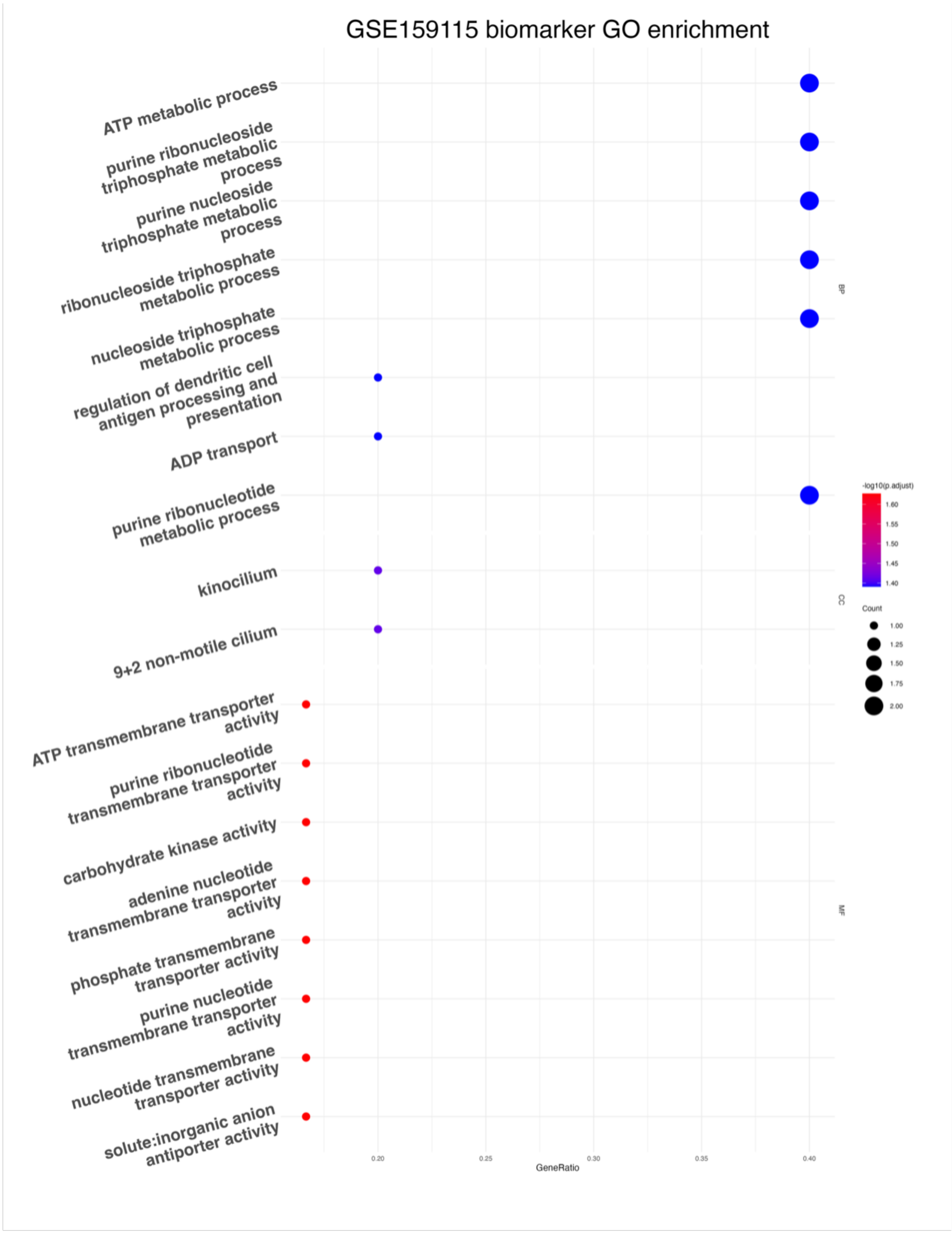

Fig. S11. GSE159115 biomarker GO enrichment.

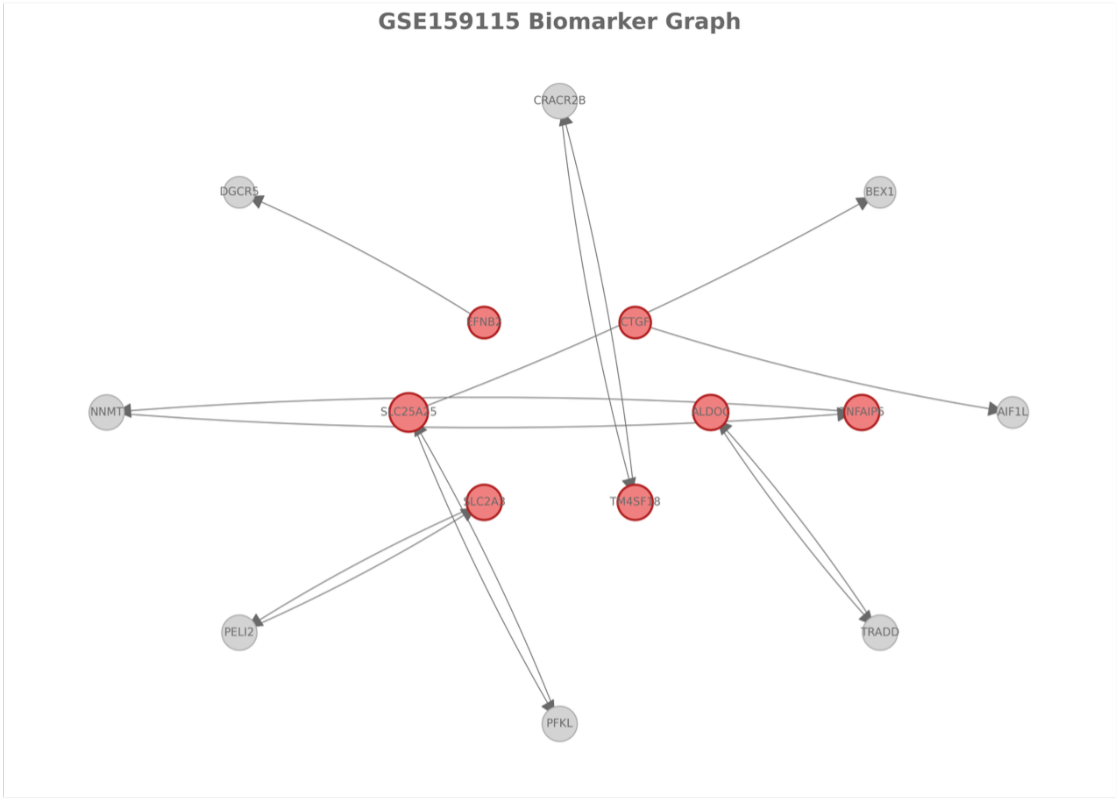

Fig. S12. GSE159115 biomarker network.

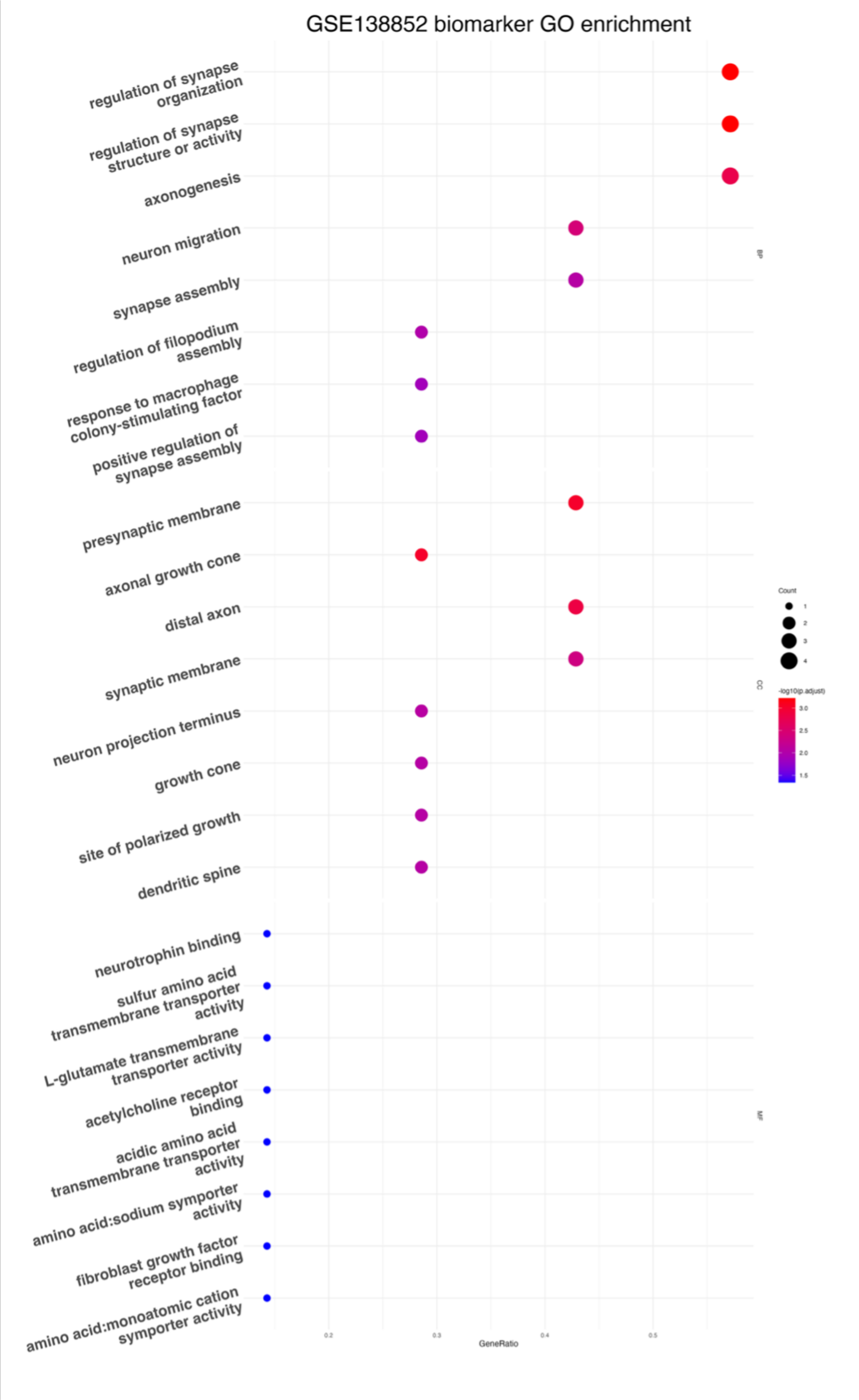

Fig. S13. GSE138852 biomarker GO enrichment.

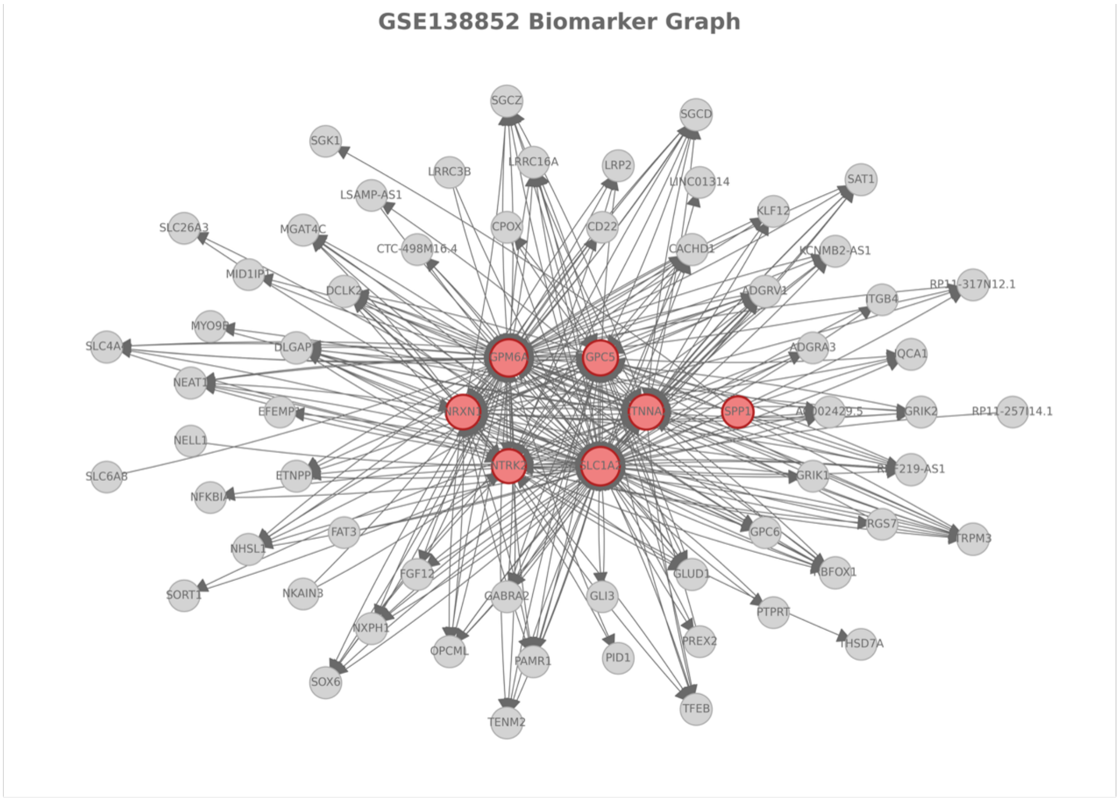

Fig. S14. GSE138852 biomarker network.

To assess whether the CSCN-ACE biomarker workflow generalizes beyond the breast tumor analysis shown in the main text, we applied the same pipeline to three additional disease settings with distinct biological architectures: ulcerative colitis (SCP259), clear cell renal cell carcinoma (GSE159115), and Alzheimer's disease (GSE138852). In all cases, candidate genes were first restricted to the top 150 DESeq2-ranked genes for the corresponding case-control comparison, after which CSCN was used to infer cell-specific causal structure and ACE was computed to retain only genes with non-zero causal effect on disease state. For SCP259, we focused on epithelial crypt/proliferative compartments and sampled 4,000 inflamed and 4,000 healthy epithelial cells. For GSE159115, we analyzed paired ccRCC tumor versus PT-B/PT-C normal kidney epithelial cells, sampling 100 tumor and 100 normal cells from matched patients. For GSE138852, we compared Alzheimer's disease and control nuclei after excluding low-confidence annotations, sampling 3,000 AD and 3,000 control nuclei. These sampling settings match the current biomarker analysis scripts in the repository and were chosen to balance disease and control groups while preserving the disease-relevant cellular context. We summarize each disease-specific biomarker panel using the retained biomarker count, representative genes, and functional enrichment relative to the broader DESeq2-only background.

S2.8.1. SCP259 biomarker validation.

The three validation datasets yielded distinct but biologically coherent biomarker panels, indicating that CSCN adapts to disease-specific regulatory structure rather than forcing a shared generic signature. In SCP259, CSCN retained 24 biomarkers, including AGR2, PI3, REG4, TFF1, PDIA3, and CEACAM5, defining a moderately sized program centered on secretory and barrier remodeling, protease inhibition, and immune-interface activity. This interpretation is supported by enriched terms such as endopeptidase inhibitor activity, peptidase inhibitor activity, and sulfur metabolism, whereas the corresponding DESeq2-only background was dominated more broadly by fatty acid oxidation and acute inflammatory-response programs. Thus, in ulcerative colitis, CSCN preferentially retained the epithelial barrier-defense and mucosal remodeling components most likely to function as direct upstream drivers.

S2.8.2. GSE159115 biomarker validation.

In GSE159115, CSCN acted much more conservatively, reducing the final panel to only 6 biomarkers, including PFKL, SLC25A25, CRACR2B, DCDC2, TM4SF18, and CD68. Although numerically small, this panel was strongly focused on metabolic bottlenecks and carbon-flow rewiring, with enrichment signals in glycolysis/gluconeogenesis, central carbon metabolism in cancer, pentose phosphate pathway, and related carbohydrate metabolic processes. This compact output is consistent with the known metabolic architecture of ccRCC and suggests that CSCN filters out widespread tumor-associated expression change while retaining a small set of rate-limiting energetic regulators.

S2.8.3. GSE138852 biomarker validation.

In GSE138852, CSCN yielded a sparse panel of 7 biomarkers, including SLC1A2, SPP1, NRXN1, CTNNA2, NTRK2, GPM6A, and GPC5. Functionally, these genes converged on neuronal and glial-interface processes, with GO enrichment concentrated in regulation of synapse organization, regulation of synapse structure or activity, axonogenesis, neuron migration, and synapse assembly. This result indicates that in the distributed multicellular setting of Alzheimer's disease, CSCN prioritizes compact structural and intercellular signaling hubs rather than broad stress-response signatures. Representative examples include SLC1A2, which is closely linked to astrocytic glutamate homeostasis **?**, and SPP1, a marker of disease-associated microglial remodeling **?**.

Taken together, these cross-dataset analyses support the conclusion that CSCN-derived biomarker panels remain disease-specific in both size and functional emphasis. The ulcerative colitis dataset produced a medium-sized epithelial remodeling program, the ccRCC dataset collapsed to a minimal metabolic-core panel, and the Alzheimer's dataset yielded an ultra-sparse synaptic and glial-interface panel. This pattern is consistent with the main-text observation that CSCN captures upstream pathogenic drivers while excluding a substantial fraction of broader DESeq2-only background signal.

References

R. Cannoodt, W. Saelens, and Y. Saeys. dyngen: a multi-modal simulator for spearheading new single-cell omics analyses. *Nature Communications*, 12(1):3942, 2021. doi: 10.1038/s41467-021-24152-2.

J. L. Close, Z. Yao, B. P. Levi, J. A. Miller, T. E. Bakken, V. Menon, J. T. Ting, A. Wall, A.-R. Krostag, E. R. Thomsen, A. M. Nelson, J. K. Mich, R. D. Hodge, S. I. Shehata, I. A. Glass, S. Bort, N. V. Shapovalova, N. K. Ngo, J. S. Grimley, J. W. Phillips, C. L. Thompson, S. Ramanathan, and E. Lein. Single-cell profiling of an In Vitro model of human interneuron development reveals temporal dynamics of cell type production and maturation. *Neuron*, 93(5):1035–1048.e5, Mar. 2017. ISSN 0896-6273. doi: 10.1016/j.neuron.2017.02.014.

P. Langfelder and S. Horvath. WGCNA: An R package for weighted correlation network analysis. *BMC Bioinformatics*, 9(1):559, Dec. 2008. ISSN 1471-2105. doi: 10.1186/1471-2105-9-559.

T. Stuart, A. Butler, P. Hoffman, C. Hafemeister, E. Papalexi, W. M. Mauck, Y. Hao, M. Stoeckius, P. Smibert, and R. Satija. Comprehensive Integration of Single-Cell Data. *Cell*, 177(7):1888–1902.e21, June 2019. ISSN 1097-4172. doi: 10.1016/j.cell.2019.05.031.

A. Vandenbon, R. Mizuno, R. Konishi, M. Onishi, K. Masuda, Y. Kobayashi, H. Kawamoto, A. Suzuki, C. He, Y. Nakamura, K. Kawaguchi, M. Toi, M. Shimizu, Y. Tanaka, Y. Suzuki, and S. Kawaoka. Murine breast cancers disorganize the liver transcriptome in a zonated manner. *Communications Biology*, 6(1):97, Jan. 2023. ISSN 2399-3642. doi: 10.1038/s42003-023-04479-w.
